# Improved dynamic imaging of multiphase flow by constrained tomographic reconstruction

**DOI:** 10.1038/s41598-021-91776-1

**Published:** 2021-06-14

**Authors:** Peter Winkel Rasmussen, Henning Osholm Sørensen, Stefan Bruns, Anders Bjorholm Dahl, Anders Nymark Christensen

**Affiliations:** 1 Department of Applied Mathematics and Computer Science, Technical University of Denmark, 2800 Kongens Lyngby, Denmark; 2 Department of Physics, Technical University of Denmark, 2800 Kongens Lyngby, Denmark; 3 Helmholtz-Zentrum Hereon, Institute for Metallic Biomaterials, 21502 Geesthacht, Germany

**Keywords:** Fluid dynamics, Imaging techniques, Hydrology, Carbon capture and storage, Computational science

## Abstract

Dynamic tomography has become an important technique to study fluid flow processes in porous media. The use of laboratory X-ray tomography instruments is, however, limited by their low X-ray brilliance. The prolonged exposure times, in turn, greatly limit temporal resolution. We have developed a tomographic reconstruction algorithm that maintains high image quality, despite reducing the exposure time and the number of projections significantly. Our approach, based on the Simultaneous Iterative Reconstruction Technique, mitigates the problem of few and noisy exposures by utilising a high-quality scan of the system before the dynamic process is started. We use the high-quality scan to initialise the first time step of the dynamic reconstruction. We further constrain regions of the dynamic reconstruction with a segmentation of the static system. We test the performance of the algorithm by reconstructing the dynamics of fluid separation in a multiphase system. The algorithm is compared quantitatively and qualitatively with several other reconstruction algorithms and we show that it can maintain high image quality using only a fraction of the normally required number of projections and with a substantially larger noise level. By robustly allowing fewer projections and shorter exposure, our algorithm enables the study of faster flow processes using laboratory tomography instrumentation but it can also be used to improve the reconstruction quality of dynamic synchrotron experiments.

## Introduction

For many years the primary technique to determine fluid flow properties of rocks was to perform classical core plug scale tests, where fluids, e.g. gases or liquids, were injected into natural porous media. The absolute permeability could then be established from Darcy’s law^1^. During the last 15 years, methods have been developed that estimate rock permeability by conducting computational fluid dynamics simulations of single or multiphase flow^2–6^. These simulations are typically based on three-dimensional pore-scale models of the rocks obtained by X-ray tomography. In recent years in situ X-ray tomography has become one of the most popular methods to directly study dynamic processes in rocks^7–9^ such as fluid flow properties^10–15^ and reactive transport in rocks^16–20^. To capture these phenomena in situ, X-ray tomography has to be performed at high spatial and temporal resolution. Therefore, most studies have been performed using synchrotron sources, which provide an extremely high X-ray beam brilliance, many magnitudes above laboratory X-ray sources^21^. Unfortunately, beamtime at synchrotron facilities is scarce and performing dynamic experiments require extensive preparation and a substantial amount of auxiliary equipment. Therefore, it is desirable to be able to perform some of the dynamic experiments using laboratory CT instrumentation.

The low photon flux of laboratory instruments leads to a compromise between image quality and the temporal resolution. Temporal resolution can be increased at the expense of image quality by decreasing scanning time. Scanning time is decreased by either reducing the exposure time of each projection, which decreases the signal-to-noise ratio or by reducing the number of projections gathered resulting in artefacts in the reconstruction^9,22^. Bultreys et al.^9^ have built a laboratory instrument for in situ microtomography, where they managed to have an impressive time scale of just 12 seconds, by using a very short exposure time combined with a reduced number of projections^9,23^.

**Figure 1:**
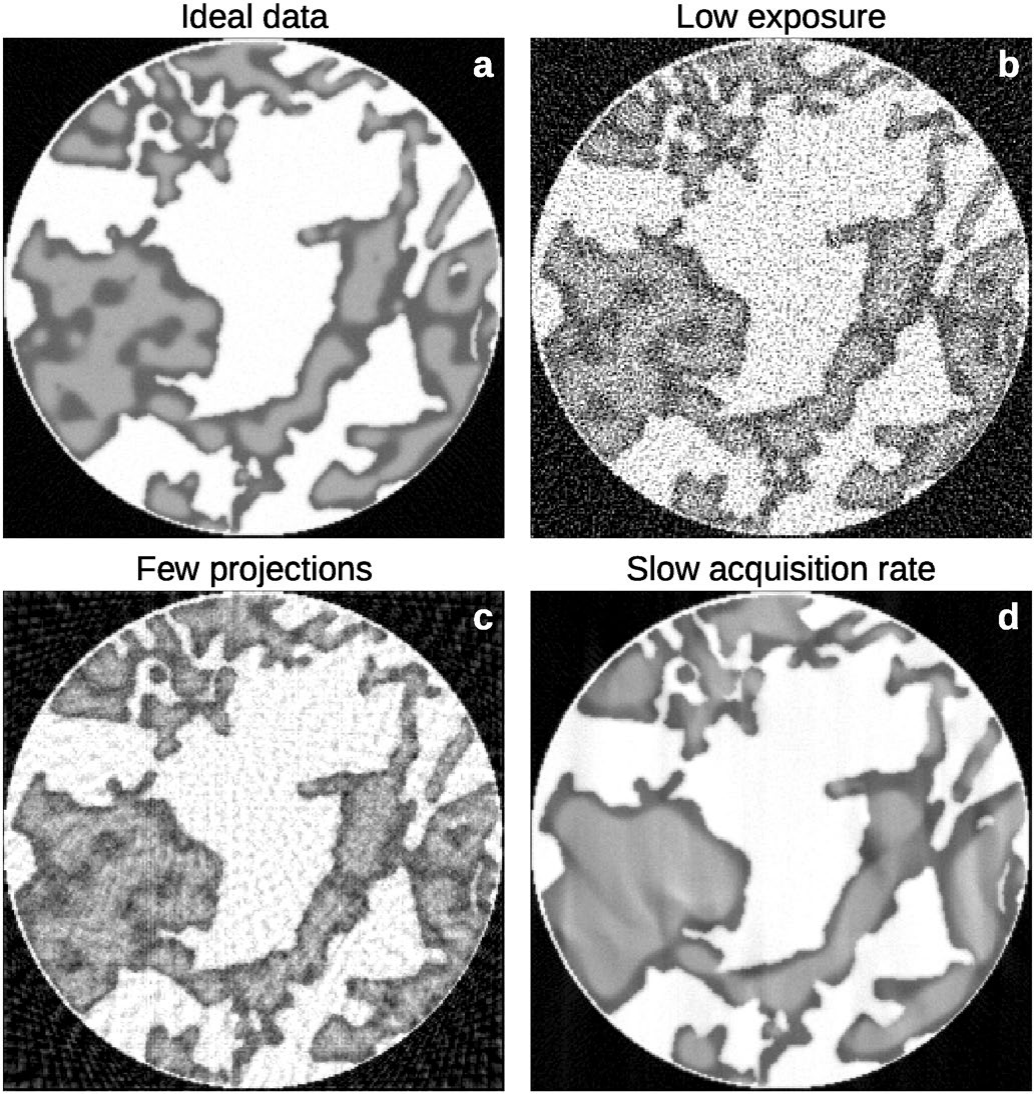
The reconstructions resulting from a tomographic experiment is highly affected by the experimental parameters. To visualise the potential effects that might occur in dynamic tomography, we have performed reconstructions of three data sets that are each limited in one experimental parameter. (**a**) The “ideal” reconstruction of the system, which is carbonate rock (white) filled with a fluid mixture of oil (dark grey) and water (light grey). (**b**) A reconstruction performed on data limited in the signal-to-noise ratio, i.e. short exposure or low X-ray brilliance. (**c**) A reconstruction from a data set with a low number of projections. (**d**) A reconstruction performed on data with high signal-to-noise (long exposure) and high number of radiographs, i.e. long data collection leading to low temporal resolution.

Figure 1 shows how three different data-deficiencies: limited exposure (high noise); a limited number of projections; and limited temporal resolution, affect the resulting reconstruction of a dynamic data set. The model system shown in Fig. 1 consists of a rock matrix (white) and two immiscible fluid phases, oil (dark grey) and water (light grey), that spontaneously separate over time. From this, we see that short exposures lead to a noisy reconstruction, few projections to line artefacts and long exposures to smeared fluid boundaries.

The most commonly used reconstruction techniques, filtered back projection (FBP) and its cone beam counterpart the Feldkamp, Davis, and Kress algorithm (FDK) are unsuited for data with the previously mentioned deficiencies^24–26^.

This is because a good reconstruction using this type of algorithm requires a rather large number of projections $N_{\mathrm {proj}}$, preferably $N_{\mathrm {proj}}\gtrapprox N_{\mathrm {pix}}\pi /2$ where $N_{\mathrm {pix}}$ is the number of detector pixels^26^. This means that thousands of low noise radiographs are needed to provide high-quality 3D reconstructions, eventually leading to high scan times – often in the order of hours^27^.

It has previously been shown that iterative reconstruction techniques perform substantially better than FBP methods when the $N_{\mathrm {proj}}$ is limited—especially when prior knowledge about the object is leveraged^26^.

Prior knowledge can be used to constrain the solution of the reconstruction algorithm to behave in a certain way. For instance, a solution can be encouraged to have a noise-free appearance by penalising the norm of the derivative of the reconstruction, which is the case in e.g. total variation regularisation^28^.

Some simple examples of using prior knowledge are non-negativity constraints and box constraints. Non-negativity stems from the fact that attenuation coefficients are theoretically always positive. This can be extended to also include an upper limit to the values allowed in the reconstruction, i.e. box constraints. Setting the upper limit requires that the largest attenuation coefficient in the sample is known.

There have been several different attempts to leverage prior knowledge to improve the quality of reconstructions through iterative methods. Lin et al.^27^ introduced a regularisation term during their minimisation similar to that of total variation regularisation. The Huber function is applied instead of the seminorm used in total variation, which preserves boundaries between different phases in the reconstruction^28^. Lin et al.^27^ tested their algorithm on a microCT data set of a Bentheimer sandstone, saturated with a mixture of brine and oil. They reported that their suggested algorithm provided a much-enhanced contrast between the reconstructed phases. Another approach was suggested by Myers et al.^29^, who limited the number of unknowns in the equation by subtracting projections recorded on the initial static system from the projections of the dynamic system. This means that only the dynamic component is reconstructed. They used Simultaneous Iterative Reconstruction Technique (SIRT) to reconstruct the difference projection data. Additionally, they encourage spatial localisation of changes between time steps, voxels within the static region are set to a fixed value and voxel values in the reconstruction are binarised, i.e. voxels are set equal to one of two values. In their case, this corresponds to either be empty or filled. Hence the reconstruction will also be automatically segmented.

That method was further developed into a Bayesian framework by Myers et al.^30^. The Bayesian framework iteratively updates a solution such that the *maximum a posteriori* estimate of the solution is found. The solution is modelled as a sum of conditional probabilities, which ensures data fidelity, the physics of the system such as noise and correlations across time. Additionally, it is possible to add terms, which constrain the dynamic solution by using a static reconstruction, that directly segments the solution and terms that regularise it. The Bayesian algorithm presented in Myers et al.^30^ is equivalent to the one presented in Myers et al.^29^ if the assumptions such as binarisation and spatial localisation are applied to the Bayesian algorithm.

Binarisation or discretisation of attenuation values is commonly used to improve the reconstruction quality for samples with only a few unmixed well-defined phases. The discrete algebraic reconstruction technique (DART) and its extension total variation regularised discrete algebraic reconstruction technique (TVR-DART), presented by Batenburg et al.^31^ and Zhuge et al.^32^, are designed for such systems.

Van Eyndhoven et al.^33^ has introduced a method, rSIRT-PWC, similar to the method by Myers et al.^29^ i.e. they separated the dynamic system into two regions – a static and a dynamic. However, they take special care to handle pixels along the border of the dynamic and static regions. The attenuation value of pixels within the static region is set to zero while the attenuation value of pixels which are either partially or fully in the dynamic region is modelled as piecewise constant functions. This assumption is appropriate for their use case where a single fluid phase is propagating through a porous media. However, it is not appropriate for two-phase fluid flow cases, where the value of a voxel might change multiple times during the dynamic process.

In this paper, we present a method that is developed with the aim to reconstruct dynamic data from two-phase fluid flow experiments, but it can be used for any dynamic experiment, where it is possible to obtain a high-quality static data set of the initial system before initiating the dynamic experiment. This could for example be a core flooding experiment where projection images could be obtained from many angles and with long exposure times before the actual flooding experiment. With this large amount of low-noise data, a detailed image of the different parts of the sample such as rock-matrix and voids could be obtained. The information gathered from a high-quality reconstruction of the static system is the crux of our reconstruction algorithm. It is used to initialise an iterative reconstruction method, which will bring the algorithm closer to a desirable solution. The reconstruction of each time is initialised by the solution of the former step. Additionally, we constrain the solution with a segmentation of the static data set.

We have investigated the performance of our proposed approach by comparison to other SIRT based algorithms as well as the commonly used filtered-back projection (FBP) algorithm. The SIRT based algorithms we compare to are simpler versions of the algorithm we have developed. We compare the results of the different algorithms qualitatively by visual inspection and quantitatively using the $\ell _2$-norm of the residual between the reconstructions and the ground truth. Furthermore, we assess the resulting image contrast by comparing histograms of reconstructed voxel values.

## Methodology

### Reconstruction

An iterative reconstruction technique is used for this work. Typically, iterative reconstruction techniques attempt to solve the linear system

1 $$\begin{aligned} b=\varvec{A}x \ , \end{aligned}$$

where $x\in \varvec{\mathbb{R}}^n$ is the reconstructed volume stored as a vector, $b\in \varvec{\mathbb{R}}^m$ is the projection data or radiographs also stored as a vector, $\varvec{A}\in \varvec{\mathbb{R}}^{m\times n}$ is the forward projection operator or the system matrix. Determining an *x* that solves the equation is typically an ill-posed problem because there is either no solution or the solution is not unique. Hence, a direct inversion of Eq. (1) is not possible^34^. We, like others, have chosen to employ the iterative reconstruction method, SIRT, because it is a robust technique and it allows us to incorporate prior knowledge when solving the linear set of equations^29,33,35,36^.

The basic principle behind the SIRT algorithm is that it uses the residual between the forward projection of the current reconstruction and the radiographs to update the solution. The update step of the SIRT algorithm is given by

2 $$\begin{aligned} x^{\left( k+1\right) } = x^{\left( k\right) } + \varvec{C}\varvec{A}^T\varvec{R}\left( b-\varvec{A}x^{\left( k\right) }\right) \ , \end{aligned}$$

where $x^{(k)}\in \varvec{\mathbb{R}}^n$ is the image obtained at the *k*th iteration, $\varvec{A}^T\in \varvec{\mathbb{R}}^{n\times m}$ is the backward projection operator, $\varvec{C}\in \varvec{\mathbb{R}}^{n\times n}$ is a diagonal matrix containing the inverse column sums of $\varvec{A}$ i.e. $c_{jj}=1/\sum _i a_{ij}$, and $\varvec{R}\in \varvec{\mathbb{R}}^{m\times m}$ is a diagonal matrix of the inverse row sums of $\varvec{A}$ i.e. $r_{ii}=1/\sum _j a_{ij}$^37^.

The starting point of the reconstruction $x^{(0)}$ can be initialised with an arbitrary vector of real numbers. However, a vector where each element has the same value (normally zero) is generally used^29,32,33^. In the present example, the rock matrix does not change during the experiment. Hence, all the voxels in the rock matrix should have constant intensity independent of the time step, and these voxels make up a large part of the sample. This means that we can initialise the first time step of the dynamic reconstruction with the high-quality reconstruction of the static sample. Additionally, for a time series of data, we suggest initialising $x^{(0)}$ for time step, *t*, with the solution of the previous time step, $t-1$, since that reconstruction is expected to be closer to our solution than a vector of zeroes.

As mentioned the rock matrix should not change during the experiment. Hence, we can also use the high-quality static reconstruction to constrain our solution. We can determine the rock matrix voxels via segmentation of the high-quality static reconstruction, which we can use to force the algorithm to keep the voxel values of the rock matrix constant. Mathematically, this operation is equivalent to projecting the right hand side in Eq. (2) onto a convex set ${\mathcal {C}}$, which only contains allowed values, using the projection operator ${\mathcal {P}}_{{\mathcal {C}}}$^38^. The projection operator is also used to apply the box constraints mentioned in the “Introduction” section, where the set would be given by

3 $$\begin{aligned} {\mathcal {C}}=\left[ \mu _{\min },\mu _{\max }\right] ^n, \end{aligned}$$

with $\mu _{\min }$ being the smallest attenuation value in the sample and $\mu _{\max }$ being the largest.

The set we project our solution onto depends on the classification of each voxel, which is derived from the segmentation. We obtain the segmentation by thresholding the static reconstruction to identify regions of either rock or fluid. Voxels with a value above the threshold are defined as rock and fixed at the expected value while voxels below the threshold might be fluid. A voxel is only defined as fluid if its value is between the attenuation values of oil and water. This leaves us with voxels which have a larger attenuation value than water but smaller than rock. Voxels within this interval cannot be uniquely assigned to either fluid or rock and are therefore subjected to regular box constraints shown in Eq. (3). Using this technique, the iterative updating step is given by

4 $$\begin{aligned} x^{\left( k+1\right) } = {\mathcal {P}}_{{\mathcal {C}}}\left( x^{\left( k\right) } + \varvec{C}\varvec{A}^T\varvec{R}\left( b-\varvec{A}x^{\left( k\right) }\right) \right) = {\mathcal {P}}_{{\mathcal {C}}}\left( {\mathrm {SIRT}}\left( x^{\left( k\right) }\right) \right) \ . \end{aligned}$$

The SIRT algorithm from the ASTRA toolbox is used because it provides highly optimised C++ and CUDA code that can be called via a Python (or Matlab) interface. This enables the use of one or more GPUs to perform the reconstructions, which is substantially more effective than using CPUs^39–41^. The projection operation ${\mathcal {P}}_{{\mathcal {C}}}$ is performed with NumPy in Python.

### Stopping criteria

A general problem associated with iterative reconstruction methods is to determine when the optimal solution is obtained. Ideally, we would like to stop iterating when the minimal $\ell _2$-norm of the residual between the ground truth and the reconstruction is reached i.e. we wish to minimise

5 $$\begin{aligned} {\text {Figure of merit}} = {}\Vert {{x^{(k)}-{\bar{x}}}}\Vert _{2} \ , \end{aligned}$$

where ${\bar{x}}$ is the ground truth. Due to noise in the projection data, the solution $x^{(k^*)}$, which minimises the figure of merit, might not be where Eq. (4) converges to as $k\rightarrow \infty$^38^.

The ground truth, ${\bar{x}}$, is not known in a real experiment, so we have to find a way to minimise Eq. (5) without being able to compute it directly. Multiple stopping rules have been proposed in the literature, however, using the normalised cumulative periodogram (NCP) of the residual, ${r}^{(k)}=b-\varvec{A}x^{(k)}\in \varvec{\mathbb{R}}^n$, seems to stop the algorithm close to the optimal solution^42–45^.

The NCP stopping rule is based on the assumption that the residual, ${r}^{(k)}$, will have an NCP similar to the NCP of white noise when Eq. (5) is minimised, because there should only be white noise left in the residual at this point. This means that all information have been extracted from the projection data and the reconstruction can therefore be terminated.

Seeing if the residual is consistent with white noise requires calculating the periodogram. A periodogram is defined as the absolute squared values of the discrete Fourier coefficients of a vector. The periodogram of the residual vector is given

6 $$\begin{aligned} \widehat{p_i} = {|}\widehat{r_i}{|}^2, \quad i=1,2,\dots ,q\quad {\text {with}} \quad {\widehat{r}}={\texttt {DFT}}({r}). \end{aligned}$$

DFT denotes the discrete Fourier transform and $q={\lceil }n/2{\rceil }$. The reason why only approximately half of the elements of $r$*r﻿* are used to calculate ${\widehat{p}}$ is because the Fourier coefficients in the power spectrum of a real vector are symmetric around the midpoint of the vector.

The normalised cumulative periodogram (NCP) is now defined as

7 $$\begin{aligned} c_j({r})=\frac{{\widehat{p}}_2+\dots {\widehat{p}}_{j+1}}{{\widehat{p}}_2+\dots {\widehat{p}}_{q+1}},\quad j=1,2\dots ,q. \end{aligned}$$

Note that the first element of ${\widehat{p}}$, known as the DC-component, is excluded from the definition such that it starts in (0, 0). The NCP value of white noise is expected to be a straight line ranging from (0, 0) to (*q*, 1). This line $c_{\mathrm {white}}$ can be used for comparison with the NCP of the residual. This can be done using the $\ell _2$-norm $r_{\mathrm {NCP}}={}\Vert {{c(r)-c_{\mathrm {white}}}}\Vert _{2}$.

A detailed description of how the NCP stopping rule is used can be found in^45^. A major benefit of this method is that it adapts to noise level in the projection data.

We terminate the reconstruction in our implementation when two iterations on either side of $r_{\mathrm {NCP}}^{(k)}$ are larger than $r_{\mathrm {NCP}}^{(k)}$. We require two iterations to prevent small fluctuations of $r_{\mathrm {NCP}}^{(k)}$ from terminating the reconstruction prematurely. It was found that $r_{\mathrm {NCP}}$ exhibited more than one minimum at low noise levels. The algorithm, therefore, iterates beyond the first detected minimum to inspect if the current is a local minimum, i.e. if there should exist a second $r_{\mathrm {NCP}}$ minimum.

The NCP stopping rule is, computationally, fairly demanding since ${r}^{(k)}=b-\varvec{A}x^{(k)}$ has to be calculated along with its discrete Fourier transform after every iteration. ${r}^{(k)}$ is calculated by ASTRA during the SIRT update step, however, only the norm of it can be retrieved which makes it necessary to calculate it explicitly after a SIRT update. CuPy is utilised to speed up the computation of the NCP via CUDA as the Fourier transform especially can benefit from parallelisation^46^. The forward projection is calculated using the ASTRA toolbox.

The implementation of the reconstruction algorithm is shown in Algorithm 1. The algorithm starts with the initialisation of the current time step using either the static reconstruction or the reconstruction of the previous time step.

This is followed by a loop where the actual reconstruction is performed. The loop is limited to $N_{\max }$ iterations to prevent the algorithm from failing to terminate. The stopping criterion is simplified as the actual implementation can handle cases where only one iteration is needed before convergence. Additionally, the implementation also continues iterating beyond the first detected minimum to ensure it is not stopping prematurely.

**Figure:**
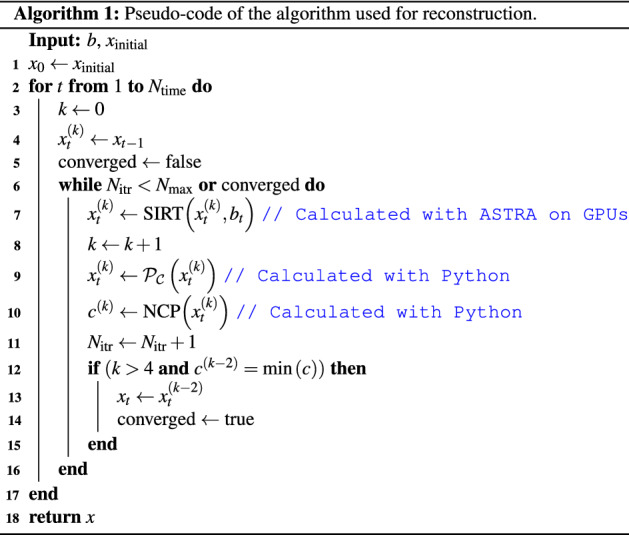


### Reconstruction algorithms used

We have chosen four versions of the SIRT algorithm and the FBP method to test the performance of our algorithm. The latter will serve for comparison as it is the most commonly used algorithm for tomographic reconstruction^26^. The SIRT algorithms are also compared to a FBP reconstruction and an ideal FBP which uses 720 projections and $\rho =0.25\%$.

The differences between the SIRT methods used are shown in Table 1. *Box constraints* means that the attenuation coefficients of voxels in the reconstruction are truncated to the minimum and maximum values present in the sample. For the present case, this means a lower limit of 0 and an upper limit of 2.5. *Initialisation* refers to initialising time step $t=0$ with the reconstruction of the static system and the remaining time steps with a reconstruction of the previous time step. Local box constraints refers to projecting the reconstruction onto the convex set ${\mathcal {C}}$ created with a segmentation of the static reconstruction as explained in the ““Reconstruction” section. This means the attenuation value of voxels identified as chalk are set to the same attenuation value as that of chalk and the attenuation value of voxels identified as fluid are confined to be within the interval of oil and water.

**Table 1:** An overview of the different approaches used for the four SIRT reconstruction algorithms that were tested.

	Box constraints	Initialisation	Local box constraints
SIRT	✗	✗	✗
SIRT-BC	✓	✗	✗
SIRT-IC	✓	✓	✗
SIRT-LC	✓	✓	✓

See text or the “Reconstruction” section for a detailed explanation.

## Results and discussion

### Comparison of the reconstructions

**Figure 2:**
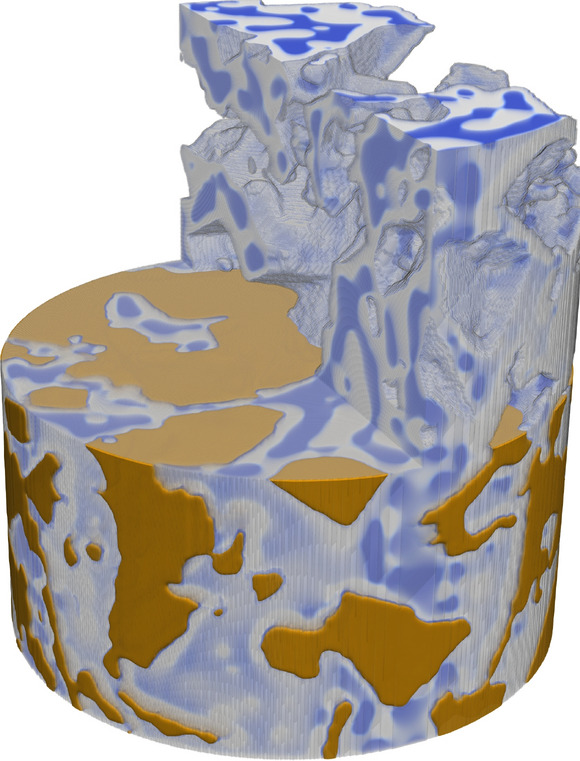
3D visualisation of the simulation. The rock matrix have been removed from the upper part of the simulation along with upper front part of the fluid phase. The rock is brown, the water is blue and the oil is light grey. This figure is created with ParaView^47^.

The method is tested on a synthetic data set that consists of a rock matrix with a homogeneous mixture of water and oil (Fig. 2), that separate over time as they are immiscible. The details of this simulation can be found in the “Methods” section. Working with simulated data enables quantitative comparisons between the different reconstruction methods since we have the ground truth. We will, from now on, use the term *residual* as the difference between the ground truth and the reconstruction unless otherwise stated. We have chosen to quantitatively examine the reconstruction methods in four ways:

- The $\ell _1$-norm of the residual between ground truth and reconstruction. This can be found in the supplementary material.
- The $\ell _2$-norm of the residual.
- The distribution of voxel values in the reconstructions.
- The distribution of the residual. This can be found in the supplementary material.

Only the voxels within the sample area are used for the quantitative analysis i.e. the air surrounding the sample is ignored.

### Visual appearance of the reconstructions

**Figure 3:**
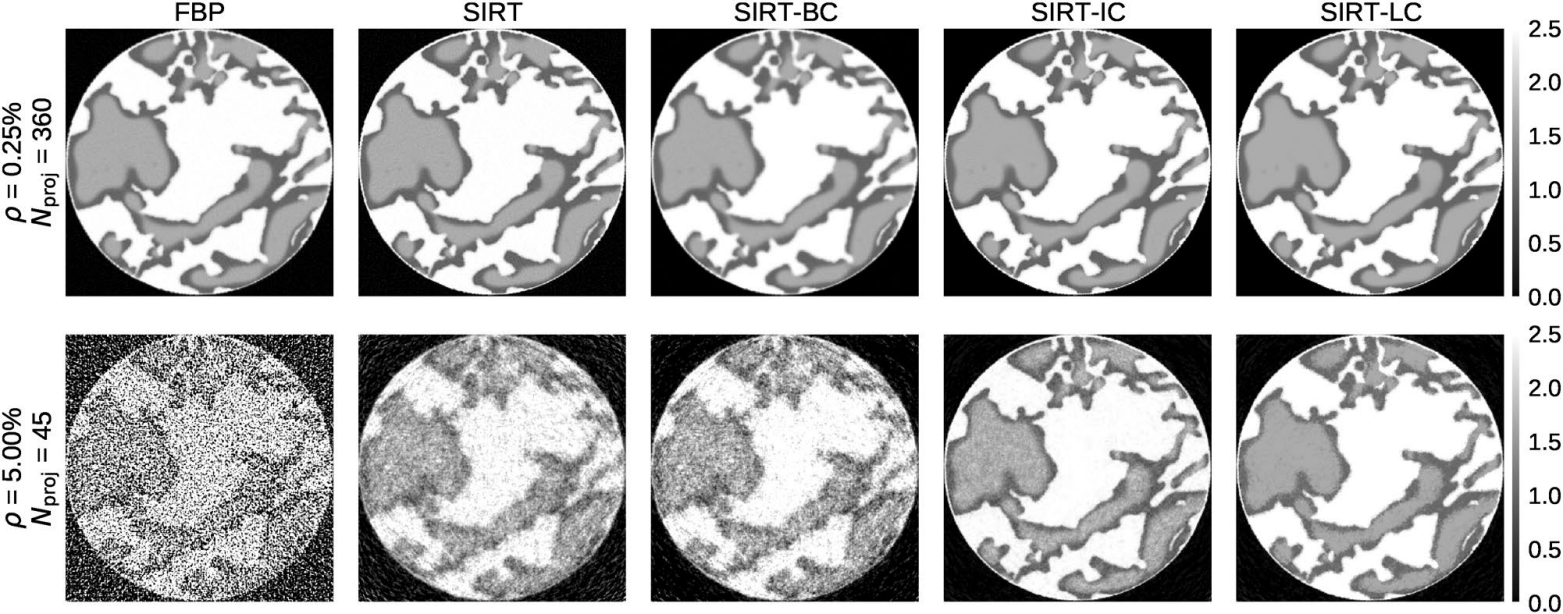
An example of the five different reconstruction algorithms for the best (upper row) and worst (lower row) data cases. Slice 171/256 at time step 51/100 is shown in the figure. Note that scale bar is truncated to [0, 2.5]. This makes the effect of box constraints present in SIRT-BC, SIRT-IC and SIRT-LC less pronounced.

Figure 3 shows two reconstruction series, one in the top row with low noise ($\rho = 0.25\%$) and a large number of projections ($N_{\mathrm {proj}}=360$) and one in the bottom row with high noise ($\rho = 5.0\%$) and a low number of projections ($N_{\mathrm {proj}}=45$). $N_{\mathrm {proj}}$ refers to the number of projections in a data set and $\rho$ refers to the relative noise level in a data set. A detailed explanation of the noise in the data sets can be found in the “Noise” section. The remaining reconstructions series can be found in Section S1 of the supplementary material. It is obvious from visual inspection that all reconstruction techniques used perform well when applied to the data set with a large number of low noise projections in the top row. In this case, the primary concern becomes computational speed.

For the other extreme, we have a data set with high noise (5%) and few projections (45), shown in the bottom row of Fig. 3, a significant difference is found in the obtained image qualities. Here the FBP reconstruction becomes very noisy. Almost to the point where it is impossible to differentiate between the two fluid phases. SIRT and SIRT-BC perform similarly, which indicates the addition of box constraints in SIRT-BC does not improve the reconstruction significantly. A major improvement is found when the reconstruction is initialised using the high-quality static data as described in the Reconstruction, which can be seen for the SIRT-IC and SIRT-LC reconstructions. The fluid phases are clearly visible using both, but SIRT-IC exhibits a fair bit of noise, which is eliminated by the local box constraints used in SIRT-LC.

### $\ell _2$-norm of the residual

**Figure 4:**
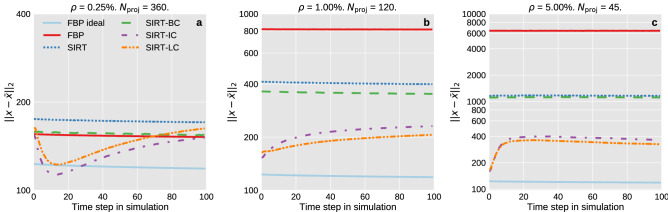
The $\ell _2$-norm of the residual as function of the time step for the three different cases of noise and number of projections. The ideal FBP reconstruction ($\rho =0.25\%$, 720 projections) is shown for comparison. Notice that the y-axis range is different on the three plots.

The performance of the algorithms has been quantified by calculating the $\ell _2$-norm of the residual between the reconstructions and the ground truth for each time step in the simulation. This has been plotted as a function of time in Fig. 4. The figure confirms that all algorithms provide good and similar results for data set reconstructed using the low noise $\rho =0.25\%$ and a high number of projections, 360. Noticeably, they all perform almost as well as the FBP reconstruction with 720 projections and $\rho =0.25\%$, the data set that represents a reconstruction under “ideal” conditions. It is apparent that FBP solution quickly deteriorates as noise increases and the number of projections is reduced. The same is partly true for SIRT and SIRT-BC, but it is not as significant. The addition of box constraints does improve the $\ell _2$-norm of the residual noticeably. However, this effect becomes increasingly less pronounced as the data degrades. The addition of initialisation substantially improves the reconstruction when the data quality degrades.

The $\ell _2$-norm for both SIRT-IC and SIRT-LC vary across time due to the initialisation which links the current time step with the previous. Both reconstructions initially improve slightly in the best data case after which their performance slightly degrades. This behaviour gets less pronounced as the quality of the data deteriorates. We performed SIRT reconstructions on a special data set where the simulation was frozen such that the first time step in the simulation was repeated for all time steps. The noise in each time step is unique. This was done to ensure that the deterioration of performance seen in Fig. 4 of SIRT-IC and SIRT-LC across time is not because the algorithms diverge. This test showed that SIRT-IC and SIRT-LC improves across time. The results from this test can be found in Fig. S8 in the supplementary material.

### NCP stopping criteria and convergence

**Figure 5:**
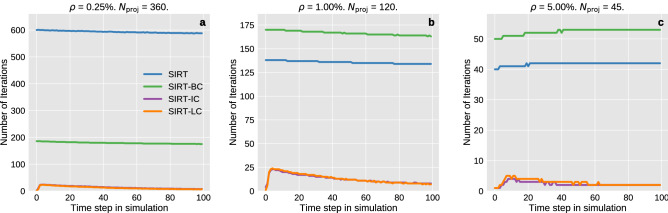
The number of iterations required in each time step before the NCP stopping criteria is met for the three different data cases. SIRT-IC and SIRT-LC nearly coincides in all cases.

The challenge when using iterative techniques to solve the linear set of equations is to determine when the optimal solution has been obtained. Here we will analyse the performance of the NCP criteria, which is used to terminate the iterative algorithms. In Fig. 4 we observed that the $\ell _2$-norm increased for later time steps. This behaviour seems to be related to the performance of the NCP stopping rule, which terminates prematurely for low noise data. In general, the method seems less suited for low noise data. This is especially true for the initialised algorithms. The number of iterations taken before the NCP stopping rule is met for each time step is shown in Fig. 5.

A general trend for all methods is that the number of iterations needed decreases as the quality of the data decreases. This is because the residual will resemble white noise more quickly as the noise level increases. The necessary number of iterations depends more on the noise level than the number of projections. This can be deduced by examining Fig. S11 in the supplementary material which shows the iterations needed for all the iterative algorithms on all data sets.

SIRT-IC and SIRT-LC show some variability in the number of iterations required compared to the two other algorithms. Initially, we see a sharp increase in the number of iterations required which is followed by a long decay. When starting, few iterations are needed because the algorithms are initialised with a reconstruction that already has converged according to the NCP criterion. The simulation changes most rapidly for the first time steps which means more iterations are needed in this period of the simulation compared to later on where the dynamics of the simulation slow down.

**Figure 6:**
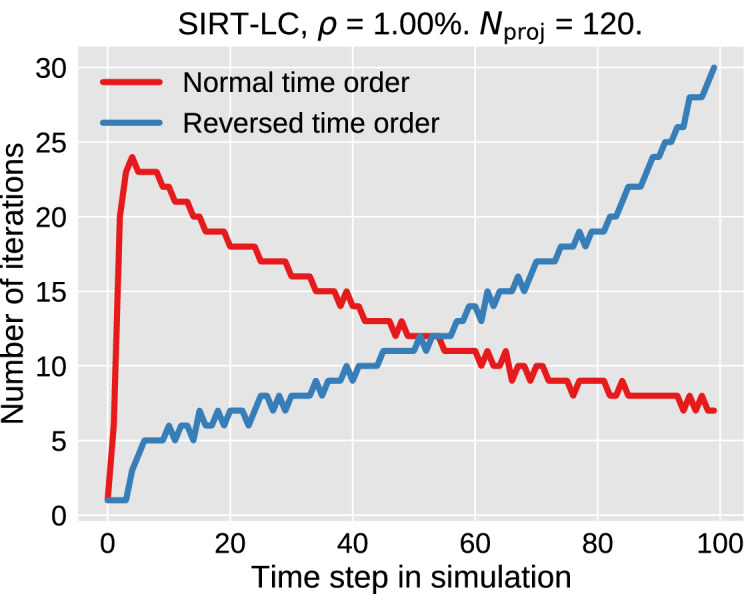
The number of iterations required in each time step before the NCP stopping criteria is met for the case with $\rho =1.00\%$ and $N_{\mathrm {proj}}=120$ for the SIRT-LC algorithm.

This is confirmed by reversing the dynamics and performing the reconstruction on this reversed data set. The iterations needed for the reversed reconstruction is shown in Fig. 6, where we see the number of iterations needed gradually increase as the dynamics of the simulation increase.

**Figure 7:**
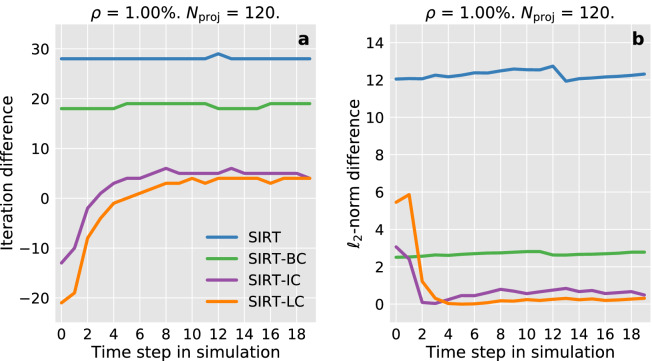
Evaluation of the performance of the NCP stopping criteria. In plot (**a**) the difference between the number of iterations used with the NCP stopping criteria and the ideal number of iterations a function of time step in the simulation. In plot (**b**) the difference between the $\ell _2$-norm of residual when using the NCP stopping criteria and the ideal $\ell _2$-norm is shown as a function of time step in the simulation.

The availability of the ground truth makes it possible to evaluate how well the NCP algorithm is at terminating at correct iteration number. This is done by comparing the solution achieved using the NCP stopping criteria with an “ideal” solution which minimises Eq. (5). The comparison consists of calculation the differences between the number of iterations used by the two stopping criteria and the difference between the $\ell _2$-norm of the two stopping criteria. This is shown for the first 20 time steps of the simulation using 120 projections with a noise level of 1.0% in Fig. 7 for the SIRT and SIRT-LC algorithms. In plot **a** we see that the NCP criteria with SIRT in general overestimates the number of iterations needed which results in a slight increase in the $\ell _2$-norm when compared to the ideal case which can be seen in plot **b** of Fig. 7. The SIRT-LC algorithm initially underestimates the number of iterations needed after which it remains fairly close to the ideal solution. In general, the NCP stopping criteria works best when the noise level is 1.0% or above and the number of projections is 120 or below. The behaviour of SIRT and SIRT-BC is very similar and the same is true for SIRT-IC and SIRT-LC.

### Histograms of voxel values

**Figure 8:**
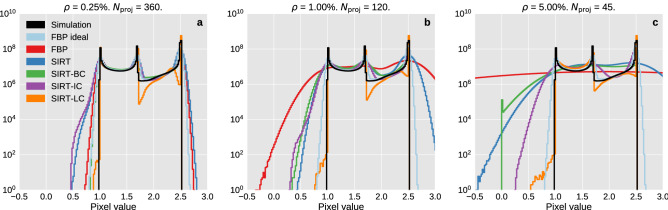
Histograms of the voxel values on a logarithmic scale of the ground truth image and the reconstructions. Plots (**a**–**c**) show histograms for reconstructions of data sets with 360 number of projections with 0.25% noise, 120 number of projections with 1.00% noise and 45 number of projections with 5.00% noise respectively.

A more direct way to compare the performance of the reconstruction algorithms is to examine the distribution of reconstructed voxel values compared to the actual voxel values in the simulation across all time steps. Some of these results are shown in Fig. 8.

The black line represents the distribution of voxel values found in the simulation. There are three distinct peaks which correspond to three phases, oil at 1.0, water at 1.7 and rock at 2.5. Values between 1.0 and 1.7 are primarily related to the mixture of oil and water, however, it can also be related to the partial volume effects at the interface between fluid and mineral, which can range from 1.0 to 2.5.

The plot in Fig. 8**a** shows the ideal data case and confirms that all algorithms give similar results for this data set as was found analysing Figs. 3 and 4. The effect of box constraints is noticeable as both FBP and SIRT have a high amount of voxels with values that far exceed the upper limit of 2.5. We also see that SIRT-IC has a tail towards 0 in plot **c** that could be a result of the limited amount of iterations used by the algorithm for that specific data set. Looking at the worst data case in **c** SIRT-IC and SIRT-LC are the only algorithms that keep having noticeable peaks, although SIRT-LC does appear a bit sharper. This increase in contrast fits well with the difference in visual appearance between in SIRT-IC and SIRT-LC as seen in Fig. 3.

**Figure 9:**
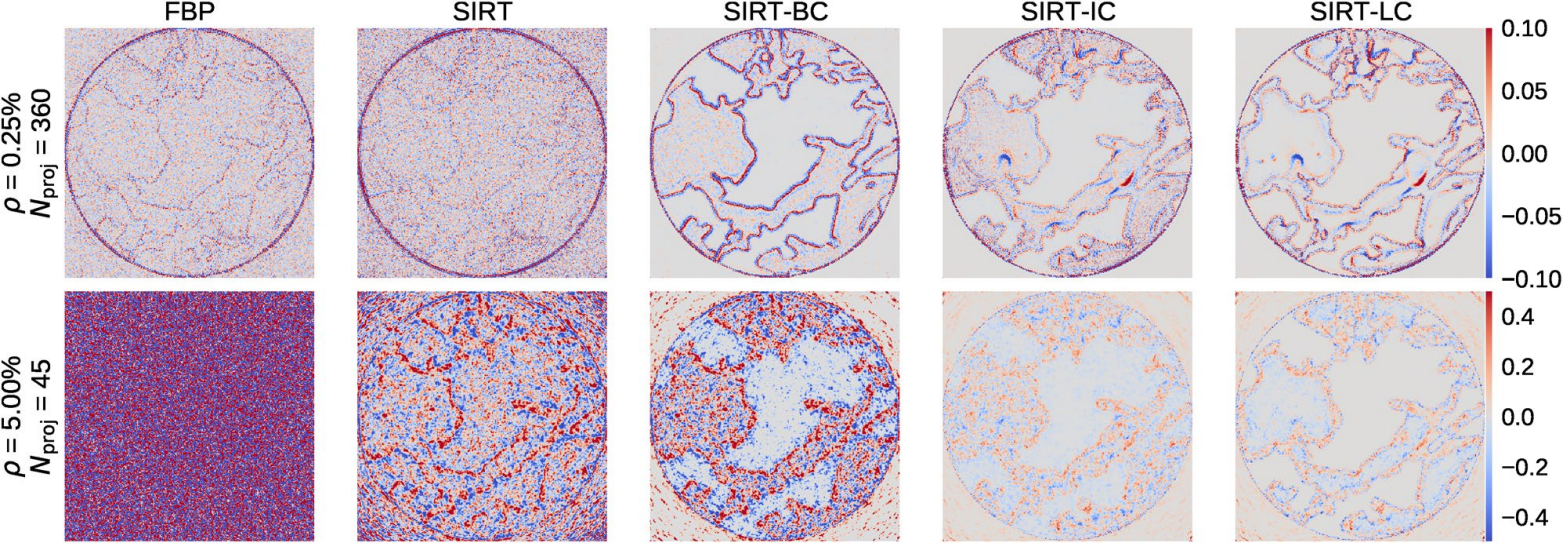
An example of the residual of the five different algorithms for the best (upper row) and worst (lower row) data cases. Slice 171/256 at time step 51/100 is shown in the figure. Pixel values are constrained to be within $\pm 0.1$ in the upper row and within $\pm 0.5$ in the lower row.

### Challenging regions in the reconstructions

In Fig. 9 the residual is shown for the best and worst data case for slice 171 at time step 51. In the upper row, we see that FBP and SIRT has most of their errors spread out compared to the remaining algorithms. SIRT-BC has most of its errors at the transition between the rock and fluid phase. In contrast SIRT-IC and SIRT-LC do fairly well in general. There are, however, large regions within the fluid phase in both of the reconstructions that are either overestimated or underestimated. This is again caused by the underestimation of iterations needed.

The bad data case shows there is no discernible area which the FBP fails to reconstruct, unlike the SIRT algorithms where there is a definite structure in the plots. SIRT-IC and SIRT-LC still handle the reconstruction fairly well with SIRT-LC being a bit more smooth.

### Global performance of the algorithms

**Table 2:** Table of the $\ell _1$- and $\ell _2$-norms for the best and worst data case.

	$N_{\mathrm {proj}}=360$ $\rho = 0.25\%$		$N_{\mathrm {proj}}=45$ $\rho = 5.00\%$	
	$\ell _1 \left( \cdot 10^{7}\right)$	$\ell _2 \left( \cdot 10^{3}\right)$	$\ell _1 \left( \cdot 10^{7}\right)$	$\ell _2 \left( \cdot 10^{3}\right)$
FBP	4.13	1.51	179.59	63.21
SIRT	4.76	1.70	32.49	11.49
SIRT-BC	2.78	1.54	25.77	11.06
SIRT-IC	2.74	**1.34**	8.68	3.69
SIRT-LC	**2.31**	1.43	**6.25**	**3.34**

Bold numbers indicate the best performing algorithm.

The $\ell _1$-norm and $\ell _2$-norm of the residual for the entire 4D reconstruction is shown in Table 2. The table shows that SIRT-LC is superior to the other algorithms in the bad data case and slightly inferior to SIRT-IC for the best data case for the $\ell _2$-norm. This was also expected based on Fig. 4 where the values for SIRT-IC are below the values of SIRT-LC. A table of all data cases is available in the supplementary material where it is seen that SIRT-LC is the best algorithm in general.

## Conclusions

We have developed a reconstruction algorithm for dynamic tomography, based on SIRT. Our algorithm targets experiments where it is possible the collect an initial high-quality tomography data set before the dynamic experiment is initiated. The reconstruction of the static system is used to initialise and constrain the reconstructions of the dynamic data via a segmentation of the static system in order to strongly regularise the solution. Additionally, we use the NCP stopping criterion to optimise the number of iterations used. We have shown using simulated data that this procedure significantly improves the quality of the reconstruction of data with a minimum number of projections and high noise levels to a point, where it is comparable to an ideal traditional reconstruction even when using poor data.

## Methods

### Computational fluid dynamics simulation

We test our reconstruction algorithm using a synthetic dynamic data set as the ground truth. The data set consists of a rock matrix with a homogeneous mixture of two immiscible fluids, modelled as an emulsion of water and oil, that separate over time while being driven upward by a small body force. The separation is initially fairly vigorous, i.e. the dynamics during this period of the experiment is much faster than later in the separation process, yielding a data set that mimics an experimental two-phase fluid system.

A segmented nanoCT data set collected on a piece of chalk, a fine-grained carbonate rock, provided a realistic environment for simulating a dynamic data set. The nanotomography measurements were performed at BL47XU, SPring-8, Japan^48^, providing a voxel size of 38 nm. 1800 projections were recorded while rotating the sample 180$^{\circ }$ with an exposure time of 150 ms. The projection data were dark current and bright field corrected. The truncated sinogram, due to a smaller FOV than the sample dimension, were completed^49^ and to avoid ring artefacts in the reconstructed image stripe artefacts were reduced in the sinogram^50^ before the 3D volume was reconstructed using the GridRec algorithm in TomoPy^51^. Noise in the 3D image was reduced using our iterative nonlocal means method^52^. A cylindrical rock matrix was made by taking a subvolume of $256^3$ voxels whereafter voxels outside a radius of 124 voxels were removed slice by slice. We mirror the rock matrix along its vertical axis to allow for vertical periodic boundary conditions of the simulation domain, i.e. the resulting cylindrical volume has a diameter of 248 voxels and length of 512 voxels.

Multiphase flow simulations were conducted following the formulation of a phase-field Lattice Boltzmann method for isothermal and incompressible fluid systems as given by Fakhari et al.^53,54^ with a custom CUDA implementation. Implementation details and parameter settings, that have been used but are not essential to our findings here, are presented in Table S2 of the supplementary material.

The initial system contains a fluid mixture of equal amounts of oil and water in every wet node that separates into an equivolumetric mixture of two separate phases with a density ratio of about 4:3, a dynamic viscosity ratio of about 3:4 and a three-phase contact angle of 90$^{\circ }$ at the rock matrix interface. The differentiability of the phase-field over the course of the simulation was ensured by modelling fluid-fluid interfaces with a three voxel wide smooth transition. Snapshots of the multiphase dynamics were generated by exporting the phase-field first after running the simulation for 3000 steps and then after every additional 100 steps until 100 frames were collected that are subsequently called time step 0 to 99. The top half of the simulation i.e. the “mirrored” part was excluded from the volume used for the simulation of the tomography experiment.

The numerical value of the phase-field voxels exported from the simulation was set equal to values measured experimentally with a laboratory CT scanner in a two-phase system presented in Lin et al.^27^. However, they used a Bentheimer sandstone instead of a carbonate. Their scan was performed at 80 KeV and both the brine and the decane used to saturate the sandstone were doped with 3.5 wt% potassium iodide. Using these measured values ensures that the contrast between the different phases of the system is comparable to a real experiment. The interface between the rock and fluid was smeared using a Gaussian filter to emulate partial volume effects, i.e. voxels, which are composed of both rock and fluid. The first recorded time frame of the simulation can be seen in Fig. 2 where the rock matrix is shown in brown and the water and oil are shown in blue and white respectively. The top part of the rock matrix along with half of the fluid phase is transparent in the figure.

### Simulation of a dynamic X-ray experiment

#### Forward projection

The fluid dynamic simulation is forward projected using a parallel beam geometry with the ASTRA tomography toolbox. The forward projection operator of ASTRA does not reflect the energy spectrum of a laboratory X-ray source and can be viewed as perfectly monochromatic. Projection angles are distributed uniformly between 0$^{\circ }$ and 180$^{\circ }$ as angles between 180$^{\circ }$ and 360$^{\circ }$ are redundant when using a parallel beam setup. The detector response is modelled as perfect and with a width of 300 pixels, to ensure that the full sample width is covered.

Each time step of the simulation is forward projected independently, which mean that we approximate individual steps as static. An alternative to this would be to include the dynamics in the forward projection such that the simulation develops between each projection. This would also make it possible to account for the time it takes for the gantry to rotate the sample which is negligible in our local CT scanner but considerable in other CT scanners. Simulating the acquisition of radiographs makes it much harder to perform quantitative analysis as a time step in the reconstruction will be composed of multiple time steps in the simulation and is it therefore not done.

If we use the same geometric matrix, $\varvec{A}$, to perform the forward projection as for the reconstruction, we will commit the so-called inverse crime, i.e. that the use the exact same discretisation both ways^55^. To avoid committing the inverse crime the forward projected data are rotated with respect to the grid of the reconstruction.

The number of projections needed for a good reconstruction using standard FBP should be larger than $N_{\mathrm {pix}}\pi /2$^26^. Our detector size of 300 pixels, means that at least 471 projections are required to perform an FBP reconstruction of high quality. To be a bit conservative 720 projections are used for both the high-quality static prior and an ideal FBP reconstruction. 45, 120 and 360 projections are used for the numerical experiments, which represent experiments with a low, a moderate and a high number of projections.

#### Noise

In real experiments, the recorded projection data will be affected by noise. The data obtained from an X-ray detector can often be assumed to follow Poisson statistics, i.e. the variance of the signal is equal to the signal itself. To do this *b* have to be converted from the negative logarithmic scale to photon counts (Step 3 in Algorithm 2). The next step of the algorithm is applying noise to the rescaled data. This is done by sampling a Poisson distribution where the intensity in each detector pixel is used as the mean of the distribution (Step 4 in Algorithm 2). Since the noise operation can only be applied on integers the floor function is applied first. The noisy projection data is scaled back to the negative logarithmic scale and returned along with the noise vector *e*.

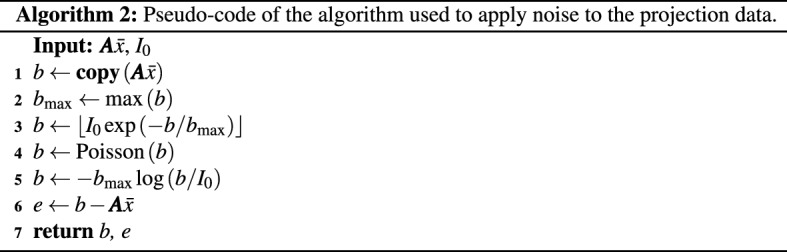



Modelling the noise as a single Poisson distribution is not entirely accurate as laboratory X-ray sources provide a wider spectrum of X-ray energies, which each differ in transmission through the sample. Hence, it in principle gives rise to multiple Poisson distributions with different means. Additionally, the X-ray detector will exhibit electrical noise which can be modelled as Gaussian noise^56–59^.

The relative noise level in the projection data is calculated using

8 $$\begin{aligned} \rho = \frac{{}\Vert {{e}}\Vert _{2}}{{}\Vert {{\varvec{A}{\bar{x}}}}\Vert _{2}} \end{aligned}$$

where *e* is the noise vector added to the forward projection of the ground truth $\varvec{A}{\bar{x}}$. We have chosen to use three noise levels which represent low, moderate and high-level noise, which is equivalent to 0.25%, 1% and 5%. These levels were based on a qualitative comparison between the noisy simulated projection data and data acquired by our local CT scanner where a noise 0.25% is generally the noise level of a high-quality scan.

### Simulated experiments

The numerical experiments were performed using the three different image noise levels (0.25%, 1% and 5%) and with three different number of projections (45, 120 and 360), i.e. nine simulated experiments will be reconstructed.

The nine different data sets are reconstructed using the five algorithms described in the “Reconstruction algorithms used” section.

## Supplementary Information

Supplementary Information.

## Data Availability

All code used for the paper along with the data sets of the attenuation coefficients are available at https://gitlab.gbar.dtu.dk/pwra/NumericalExperiments and 10.11583/DTU.c.5448594. All figures are created with Matplotlib^60^ except where noted otherwise.
