# Supplementary material for: Improved dynamic imaging of multiphase flow by constrained tomographic reconstruction
Source: Sci Rep. 2021 Jun 14;11:12501. doi: 10.1038/s41598-021-91776-1 (PMC8203785; doi:10.1038/s41598-021-91776-1)
Supplement: Supplementary file 1 — Supplementary Information. [file 41598_2021_91776_MOESM1_ESM.pdf]

Supplementary material for

Improved dynamic imaging of multiphase flow by constrained tomographic reconstruction

Peter Winkel Rasmussen[a,*], Henning Osholm Sørensen[b], Stefan Bruns[c], Anders Bjorholm Dahl[a] & Anders Nymark Christensen[a]

[*]Corresponding author.

[a]*Technical University of Denmark, Department of Applied Mathematics and Computer Science. Richard Petersens Plads, Building 324, DK-2800 Kgs. Lyngby, Denmark*

[b]*Technical University of Denmark, Department of Physics. Fysikvej, Building 307, DK-2800 Kgs. Lyngby, Denmark*

[c]*Helmholtz-Center Geesthacht, Centre for Materials and Coastal Research, Institute of Materials Research, Division for Metallic Biomaterials, Max-Planck-St. 1, Geesthacht D-21502, Germany*

Contents

Contents **1**

S1 Examples of the Reconstructions **2**

S2 ℓ_1- and ℓ_2-norms of the Residual of the Reconstruction **7**

S3 Divergence Test of SIRT-IC and SIRT-LC **9**

S4 Histograms of Residuals **10**

S5 Histograms of Voxel Values **11**

S6 Number of Iterations Used with the NCP Stopping Criteria **12**

S7 Iteration Difference Between NCP and Ideal Stopping Criteria **13**

S8 ℓ_2-Norm Difference Between NCP and Ideal Stopping Criteria **17**

S9 Examples of Residuals of the Reconstructions **21**

S10 Overall Performance of all Algorithms on all Data Sets **26**

S11 Parameters Used for Multiphase Flow Analysis **27**

S1 Examples of the Reconstructions

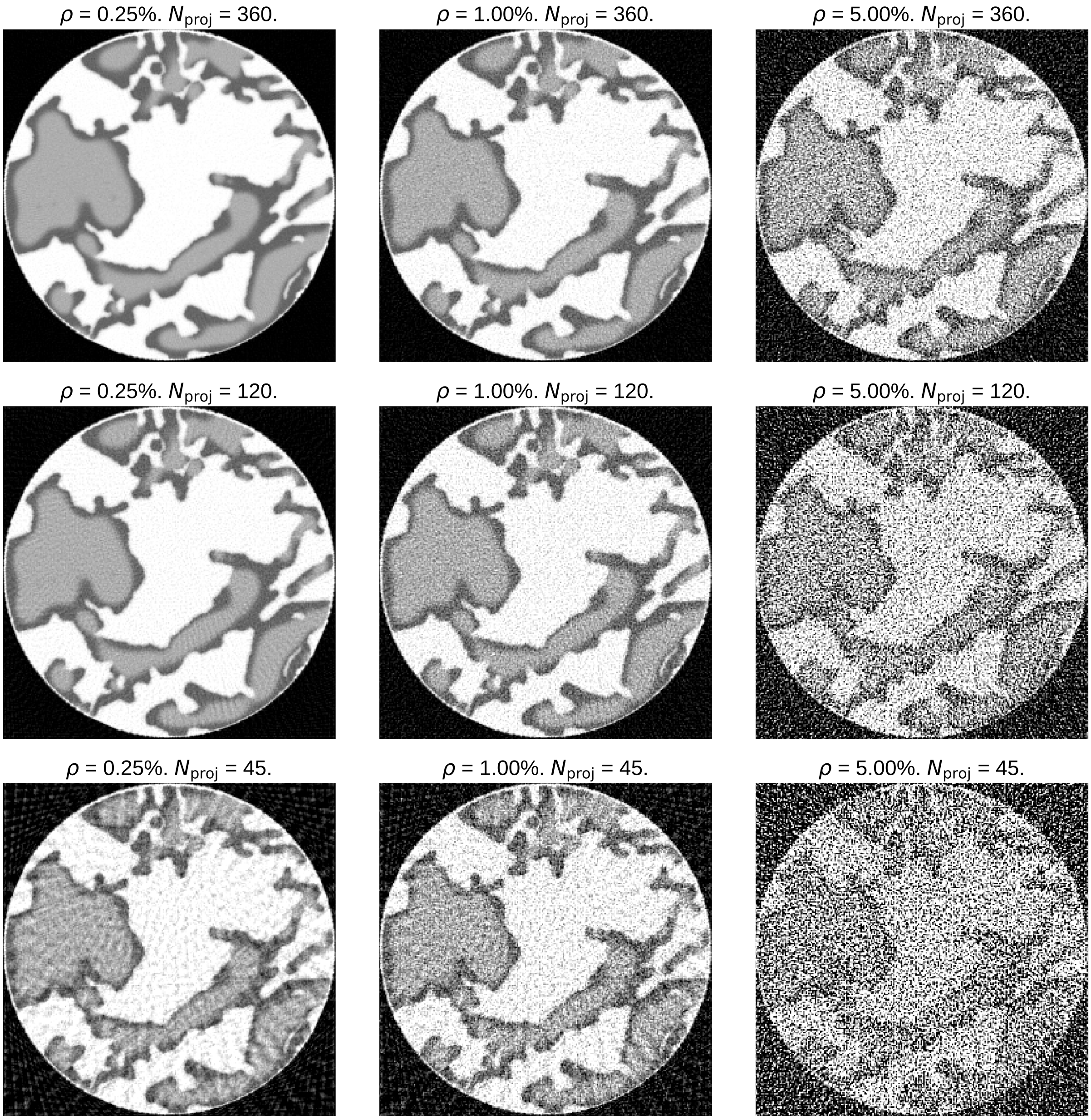

Figure S1: Examples of the reconstruction for all data sets with the FBP algorithm. Slice 171/256 at time step 51/100 is shown in the figure.

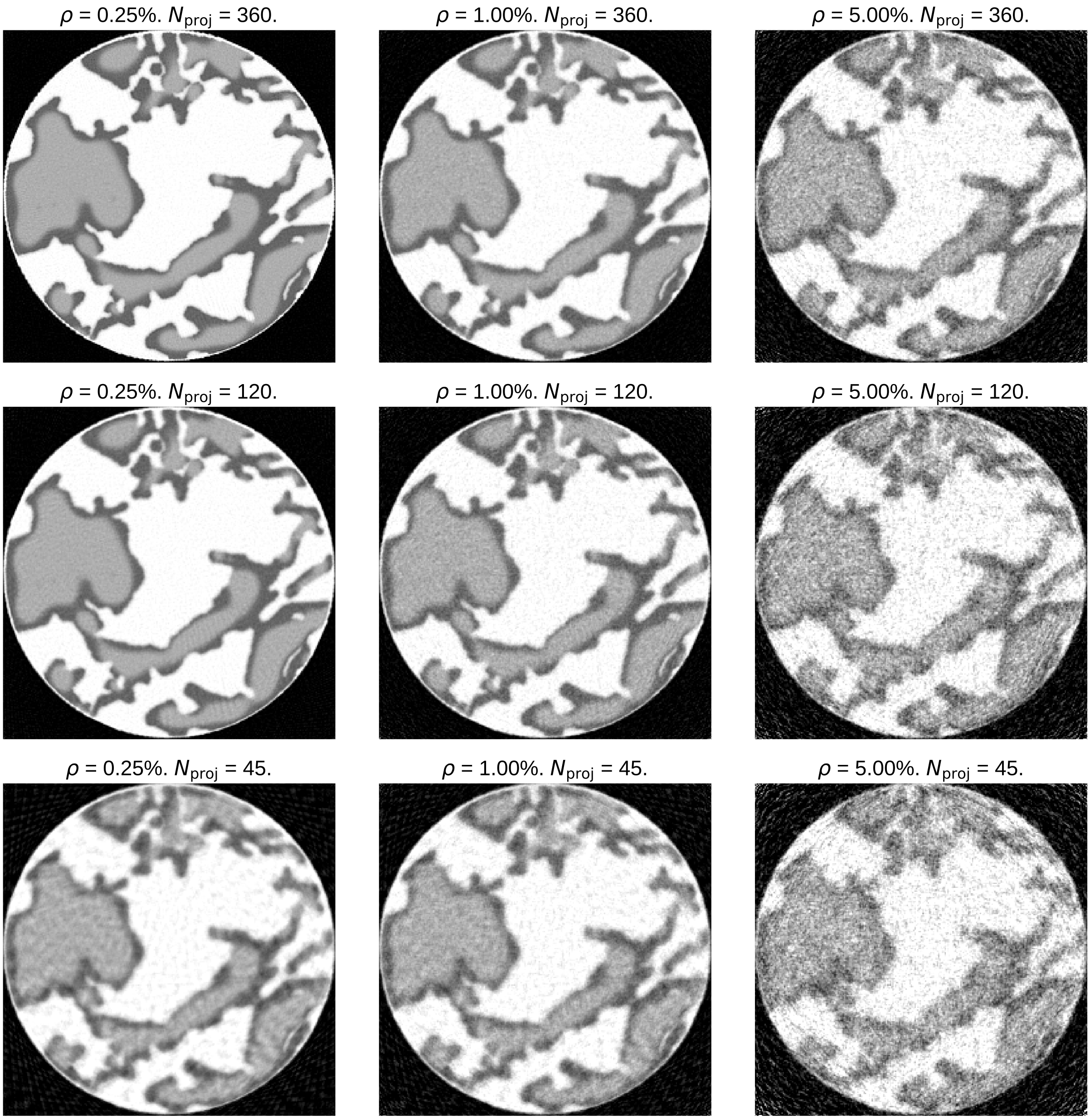

Figure S2: Examples of the reconstruction for all data sets with the SIRT algorithm. Slice 171/256 at time step 51/100 is shown in the figure.

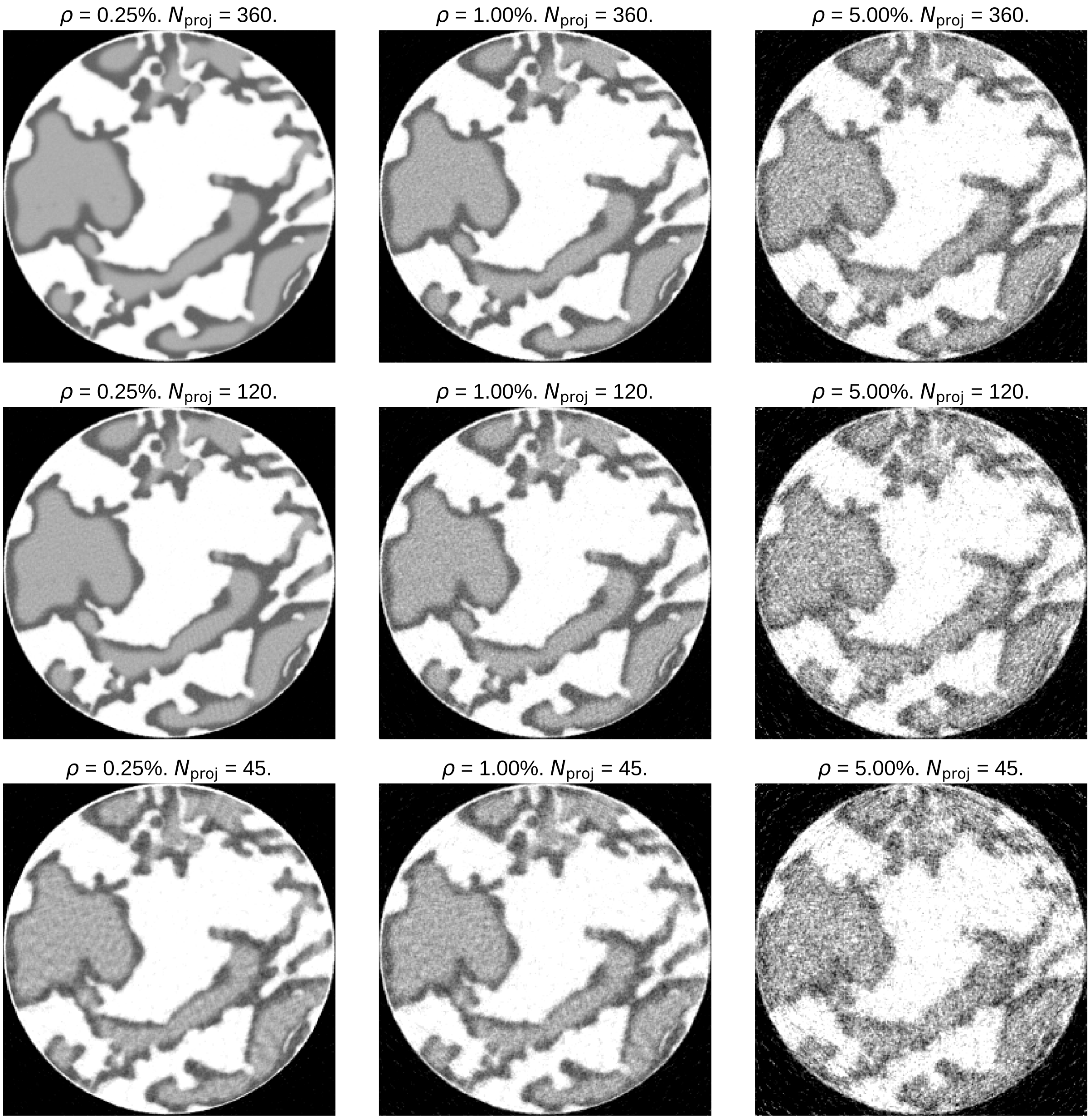

Figure S3: Examples of the reconstruction for all data sets with the SIRT-BC algorithm. Slice 171/256 at time step 51/100 is shown in the figure.

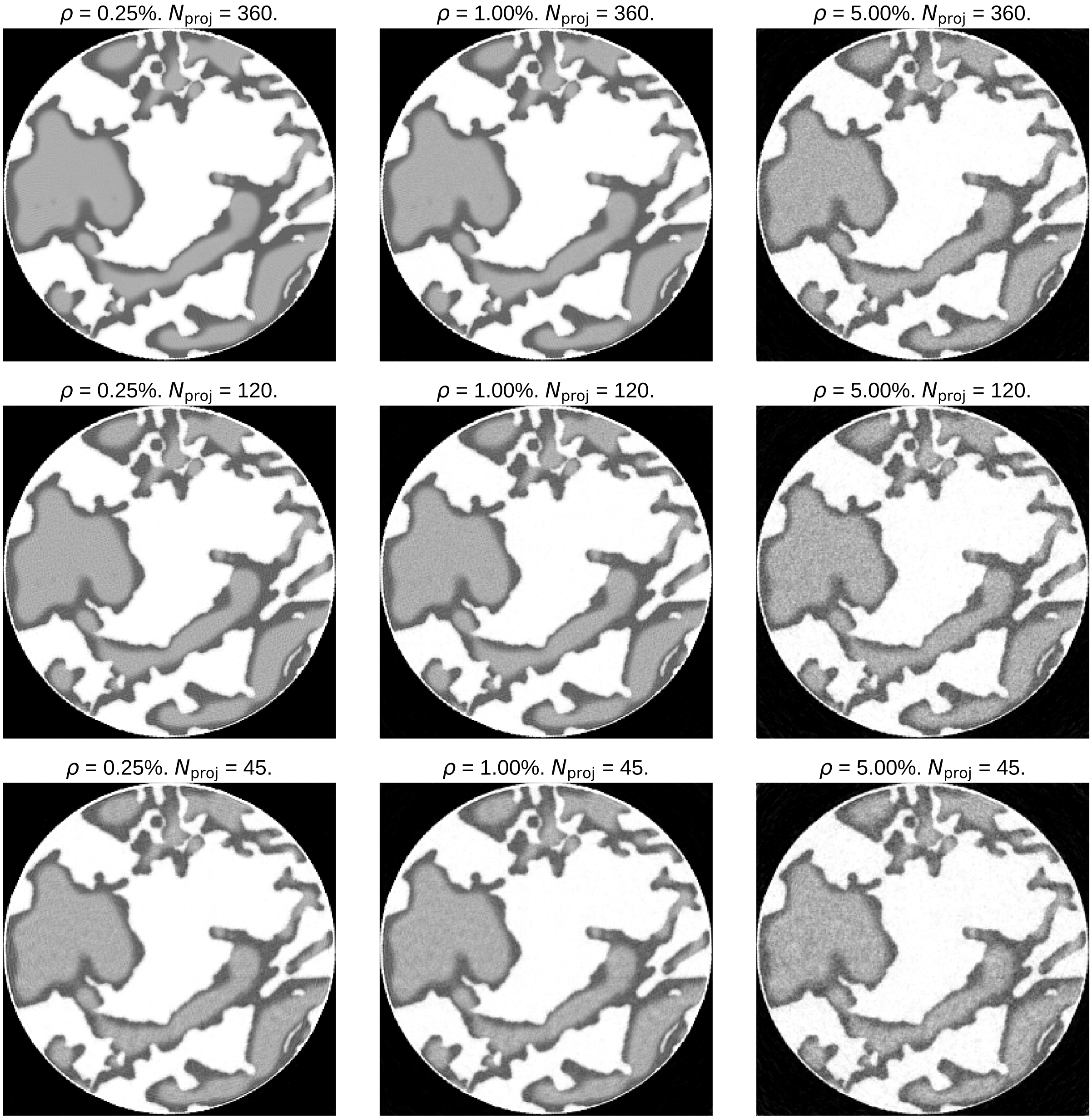

Figure S4: Examples of the reconstruction for all data sets with the SIRT-IC algorithm. Slice 171/256 at time step 51/100 is shown in the figure.

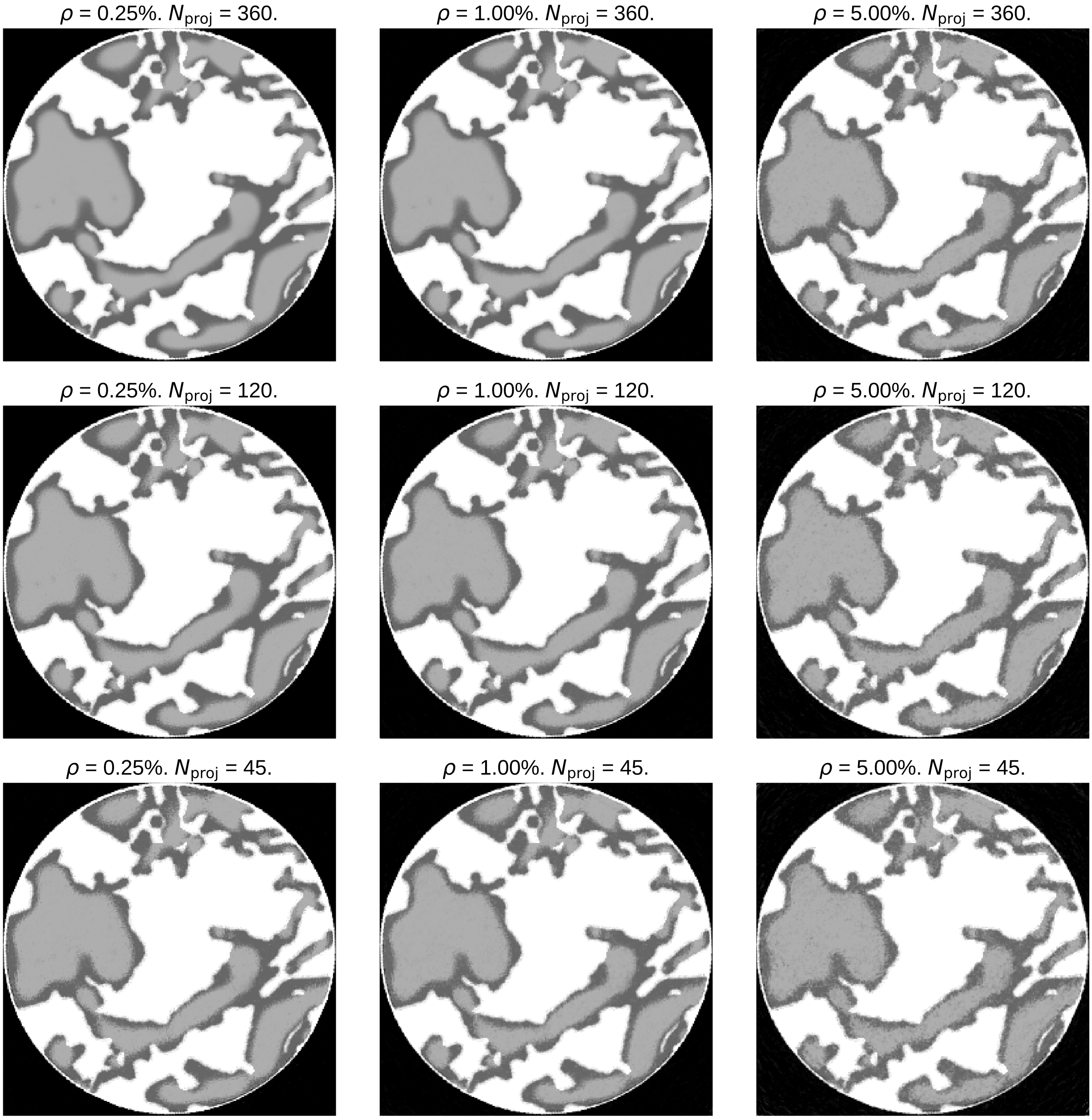

Figure S5: Examples of the reconstruction for all data sets with the SIRT-LC algorithm. Slice 171/256 at time step 51/100 is shown in the figure.

S2 ℓ_1- and ℓ_2-norms of the Residual of the Reconstruction

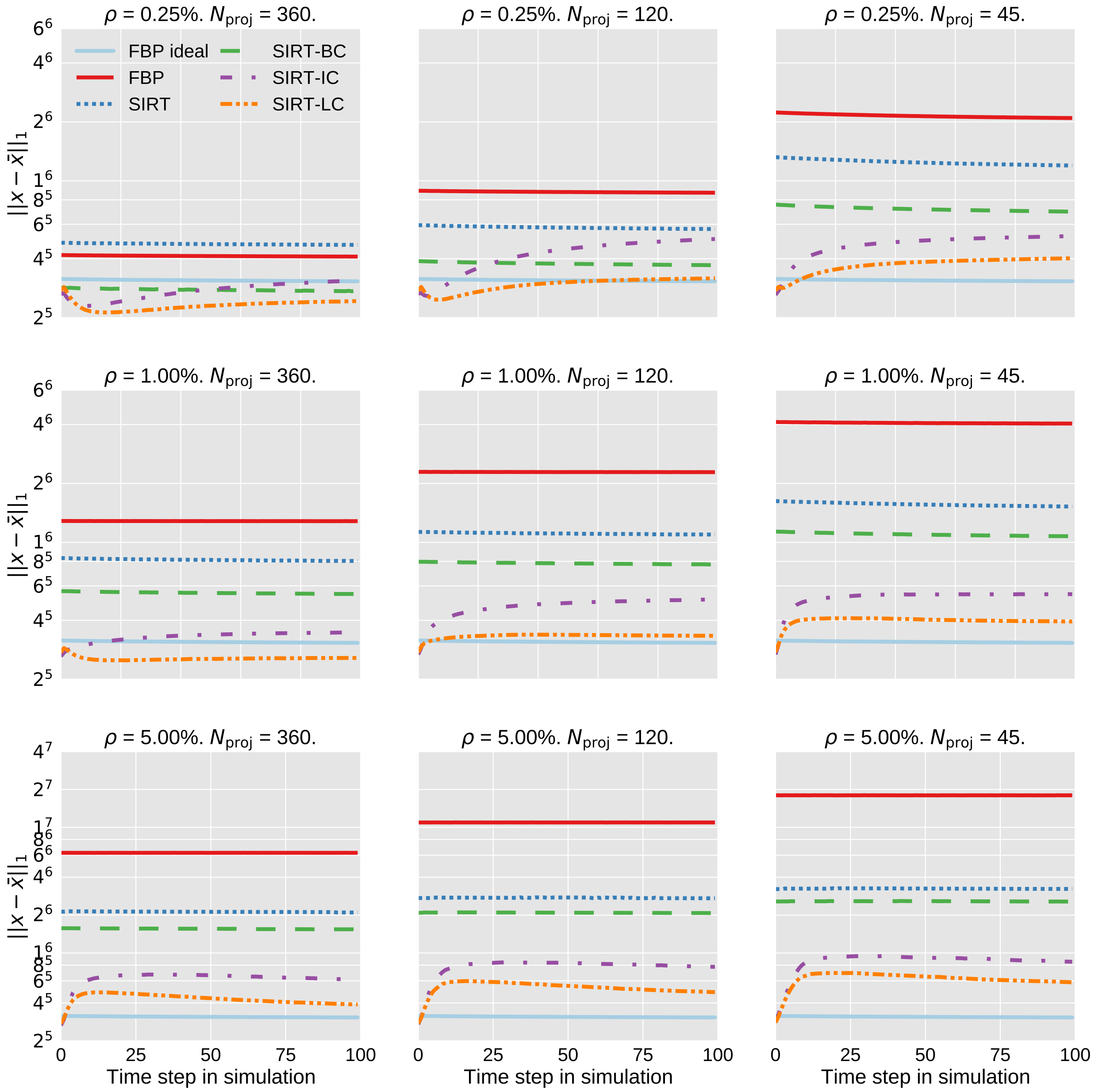

Figure S6: ℓ_1-norm the residual between the reconstruction and the ground truth for all reconstructions as a function of time step in the simulation.
The scales indicated on the vertical axis on the plots in the first column is repeated across the rows the plots are in.

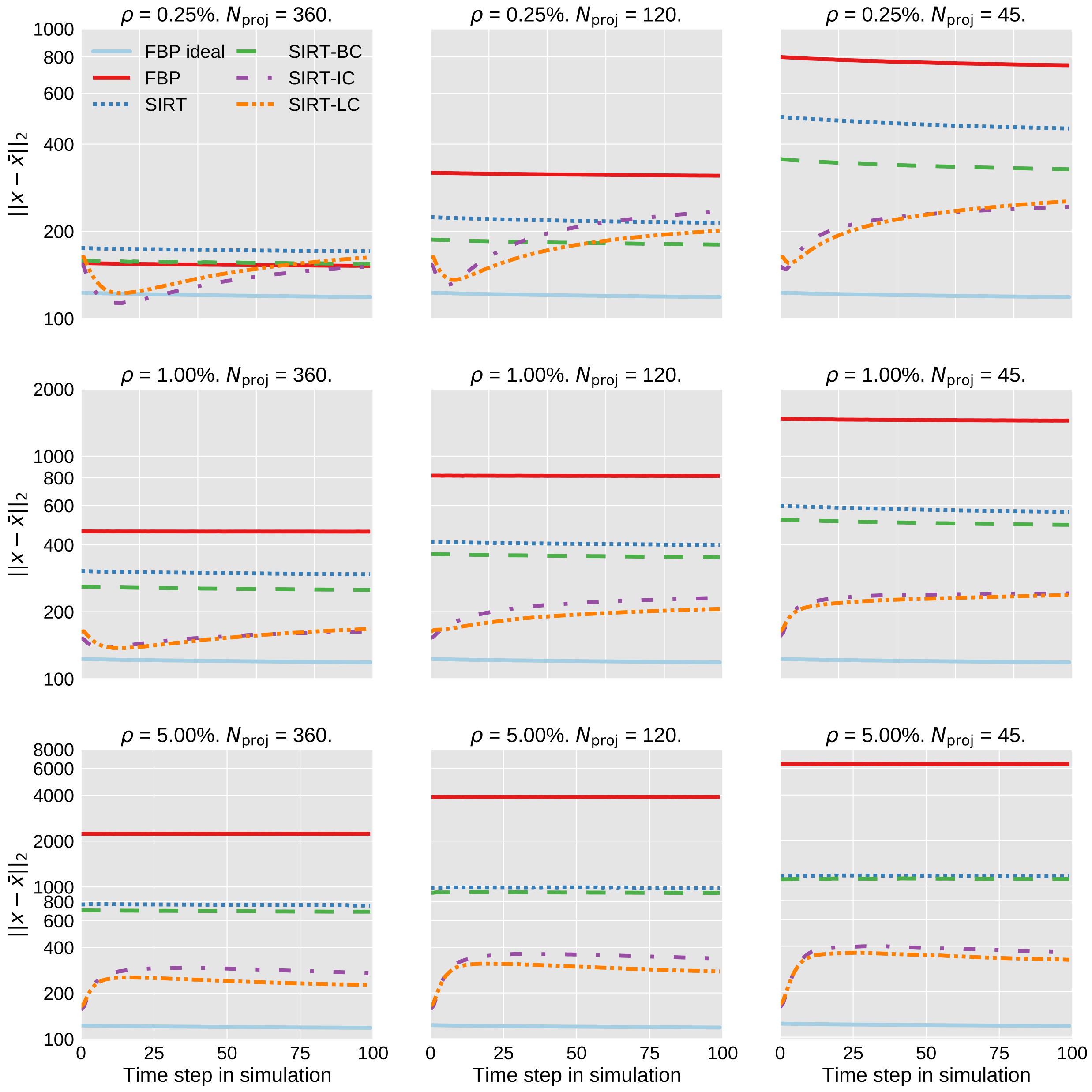

Figure S7: ℓ_2-norm the residual between the reconstruction and the ground truth for all reconstructions as a function of time step in the simulation.
The scales indicated on the vertical axis on the plots in the first column is repeated across the rows the plots are in.

S3 Divergence Test of SIRT-IC and SIRT-LC

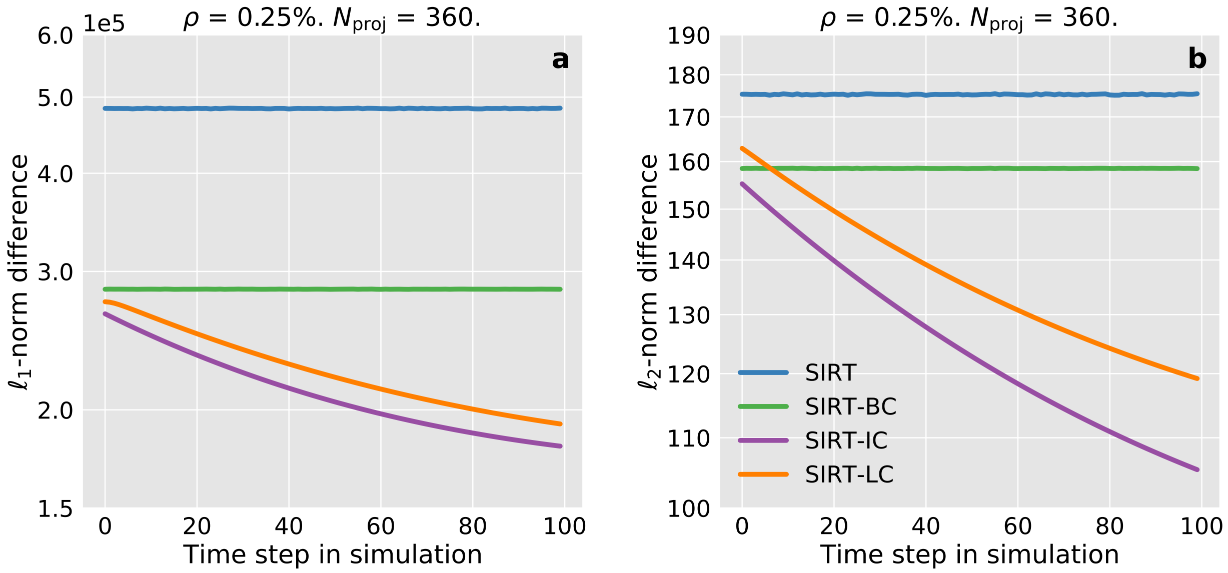

Figure S8: SIRT reconstructions performed on a frozen simulation where the first time step of the simulation is repeated for 100 time steps. The noise is unique for each time step. Figure **a** shows the ℓ_1-norm of the reconstruction as a function of time steps in the simulation and Fig. **b** shows the ℓ_2-norm of the reconstruction as a function of time steps in the simulation.

S4 Histograms of Residuals

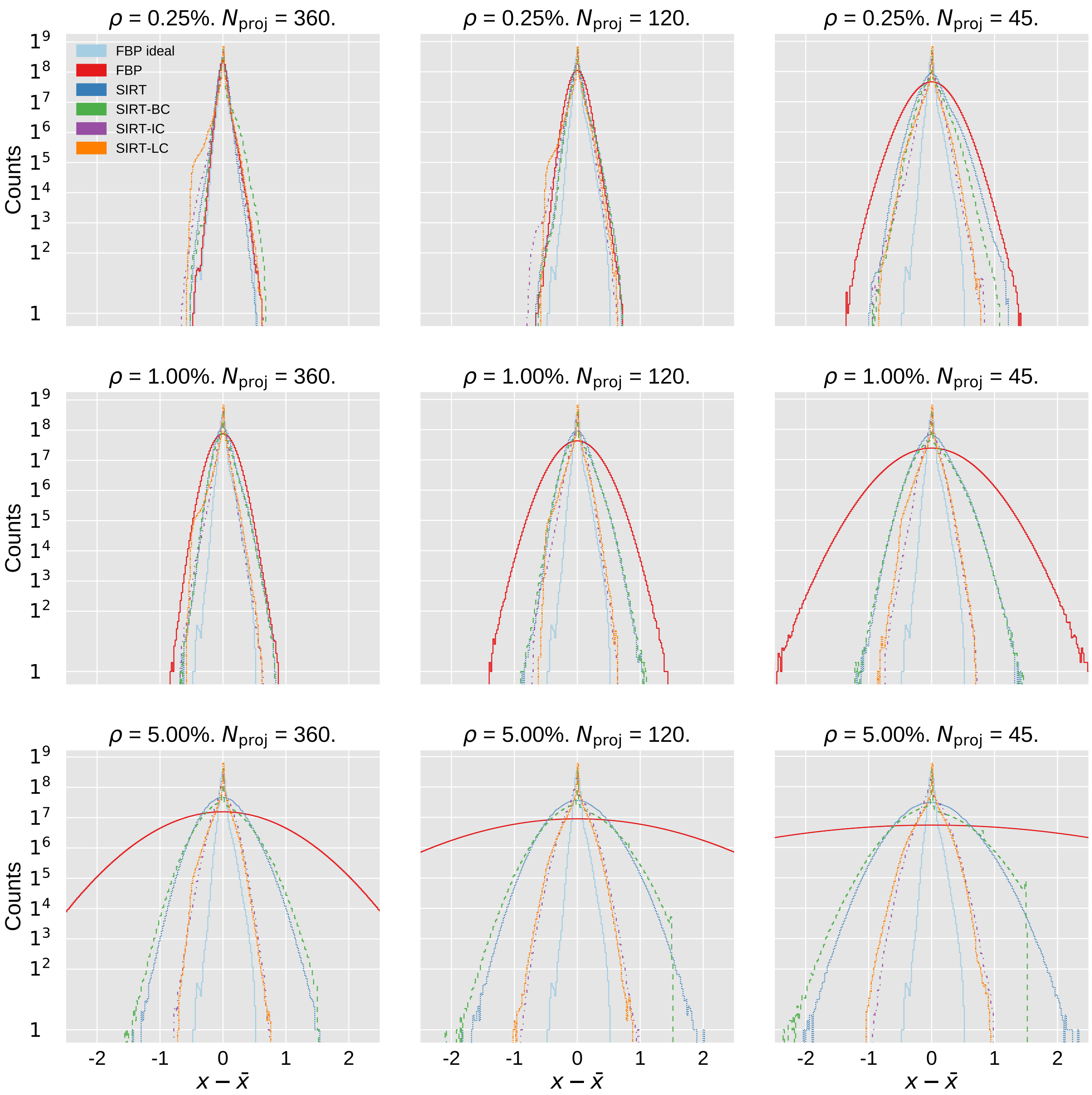

Figure S9: Histogram of voxel values the residual between the reconstruction and the ground truth for all reconstructions.

S5 Histograms of Voxel Values

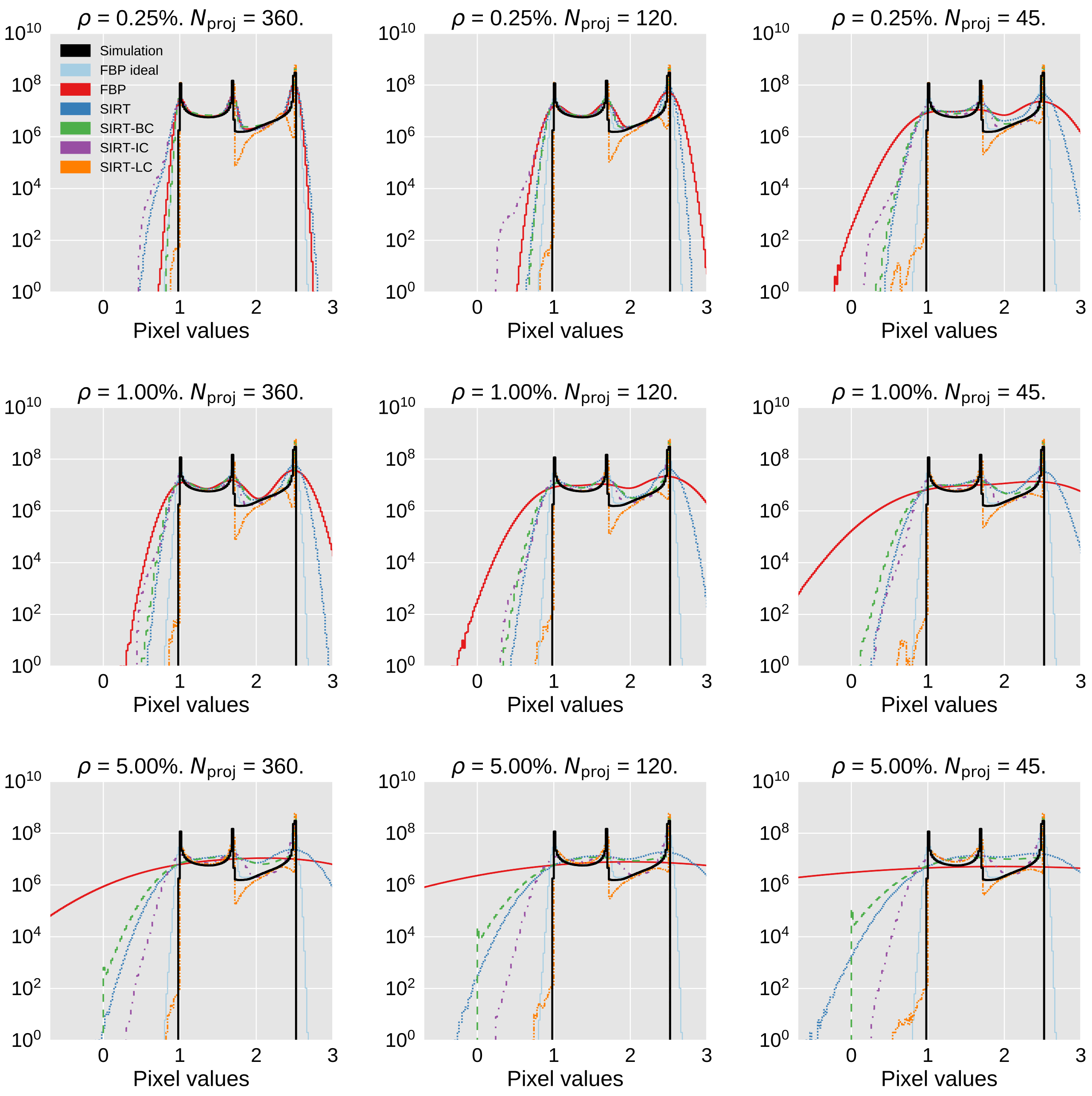

Figure S10: Histogram of voxel values for all reconstructions.

S6 Number of Iterations Used with the NCP Stopping Criteria

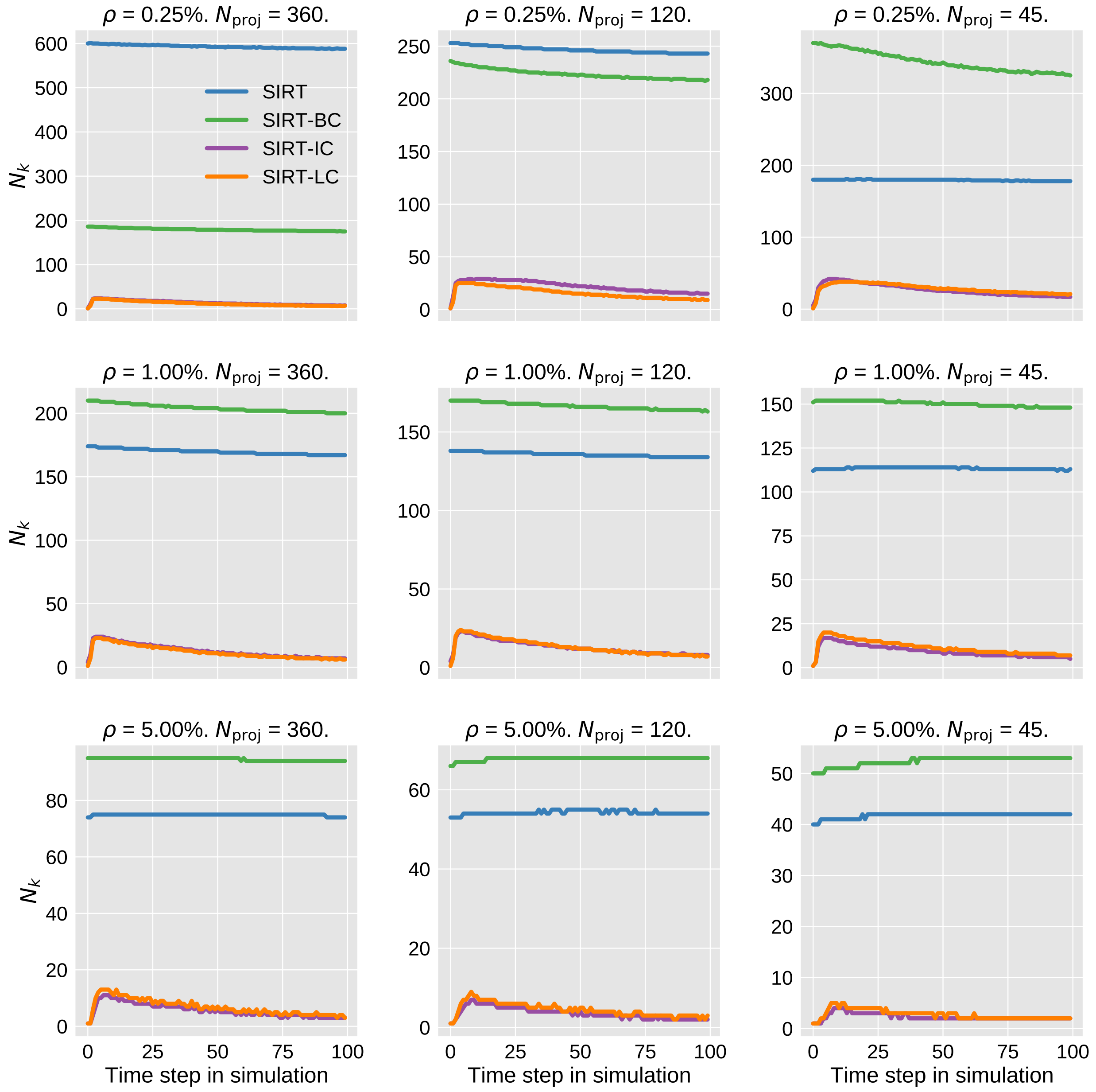

Figure S11: The number of iterations required in each time step before the NCP stopping criteria is met for for all SIRT-type reconstructions. SIRT-IC and SIRT-LC nearly coincides in all cases.

S7 Iteration Difference Between NCP and Ideal Stopping Criteria

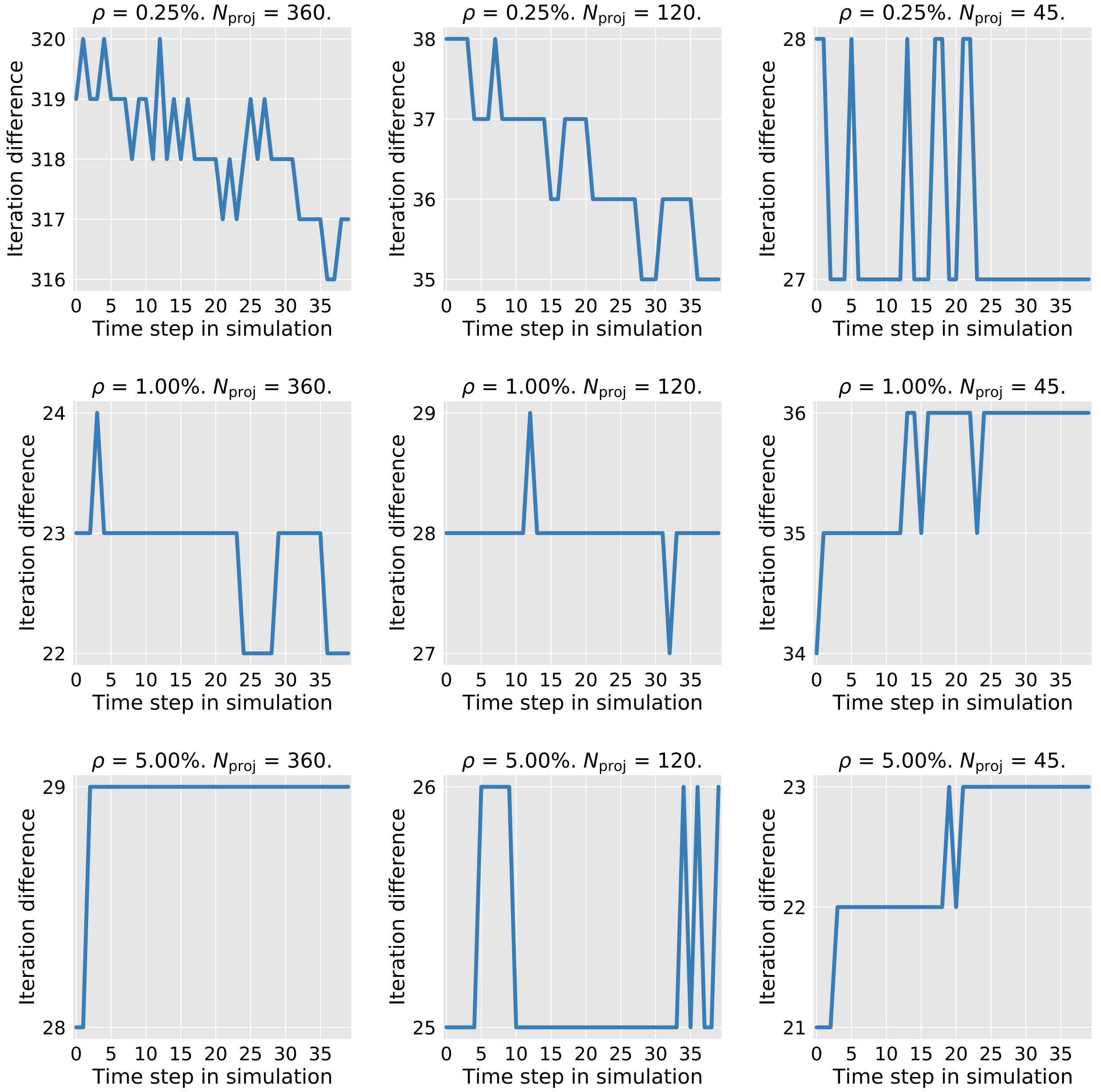

Figure S12: The difference between the number of iterations used with the NCP stopping criteria and the ideal number of iterations is shown for the SIRT algorithm as a function of time step in the simulation.

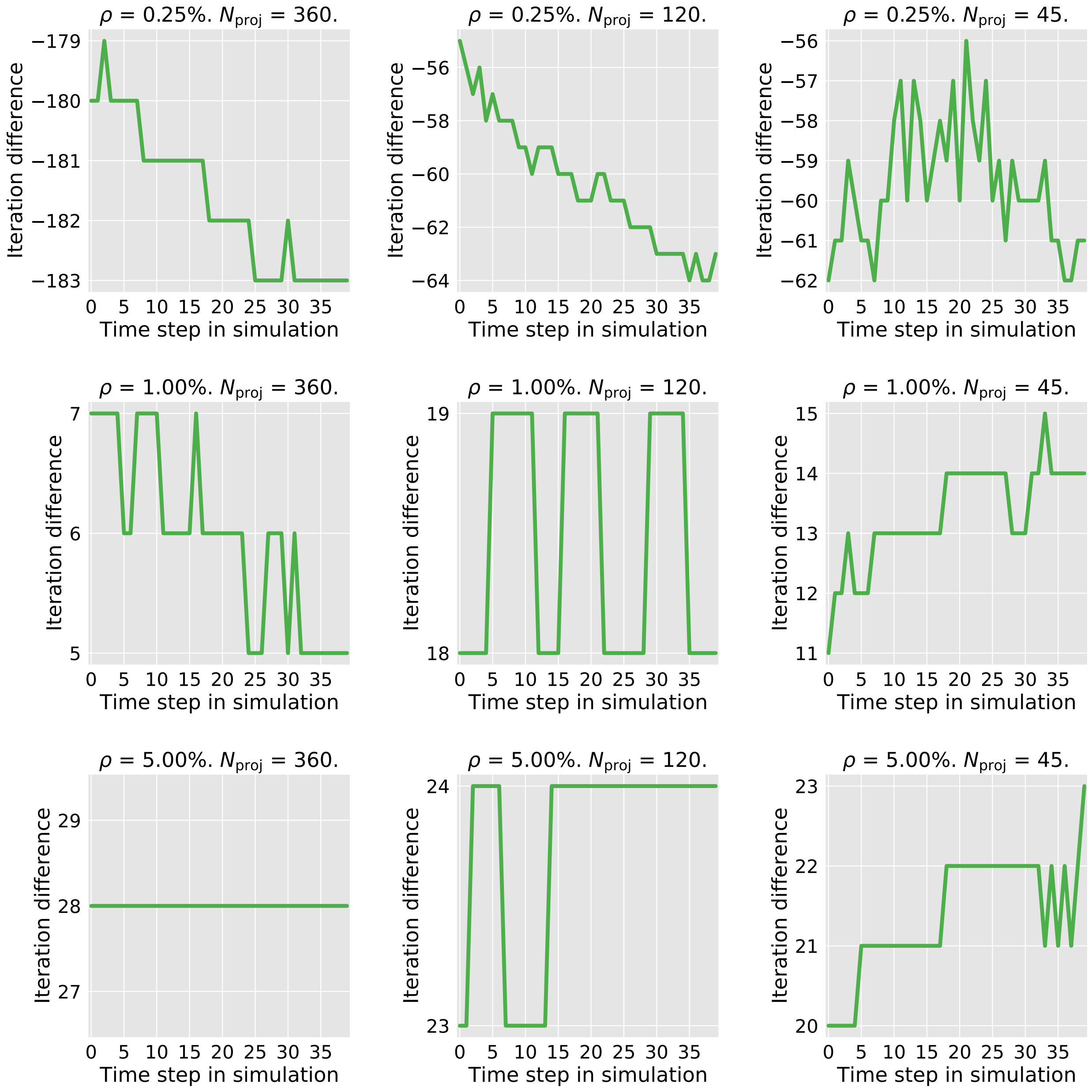

Figure S13: The difference between the number of iterations used with the NCP stopping criteria and the ideal number of iterations is shown for the SIRT-BC algorithm as a function of time step in the simulation.

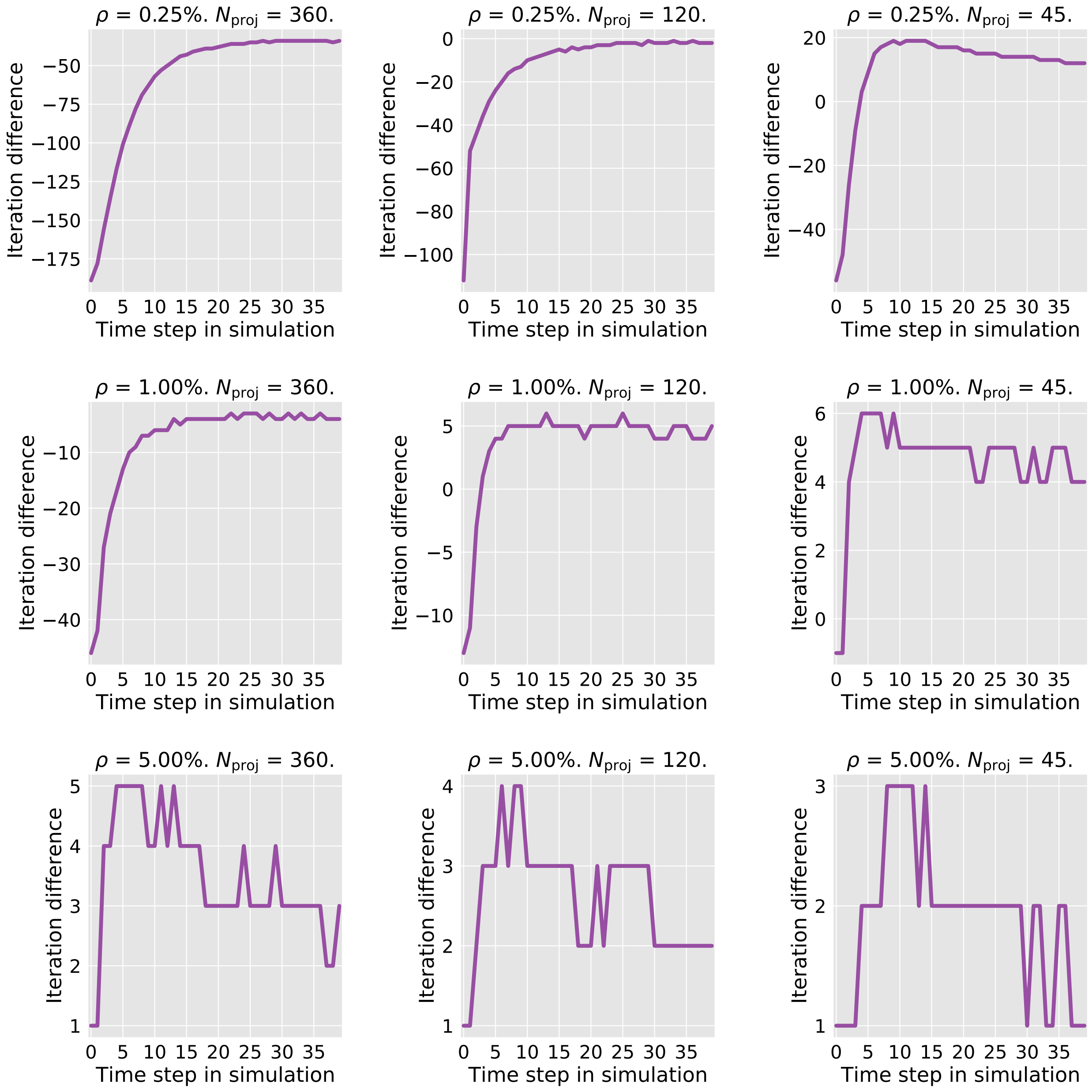

Figure S14: The difference between the number of iterations used with the NCP stopping criteria and the ideal number of iterations is shown for the SIRT-IC algorithm as a function of time step in the simulation.

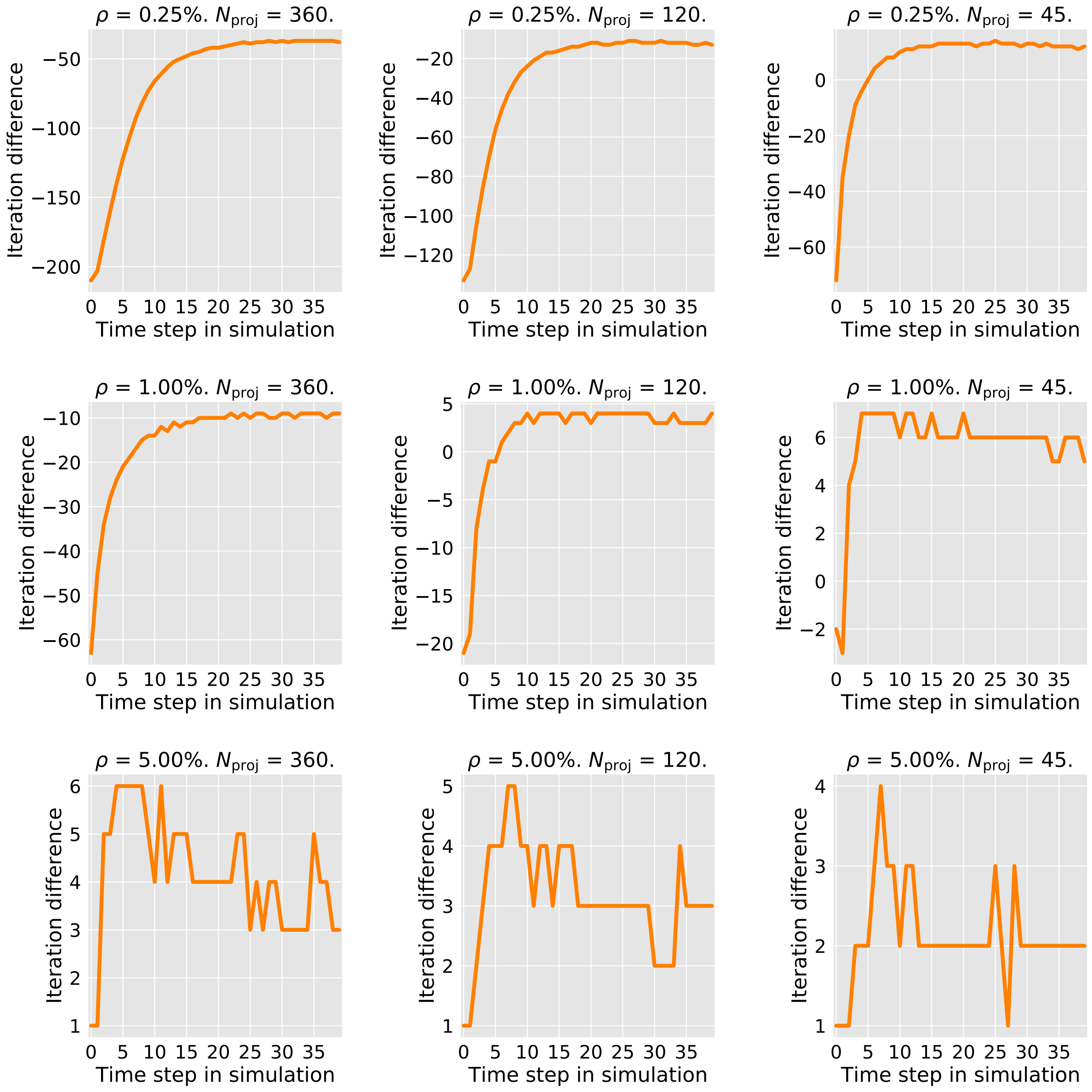

Figure S15: The difference between the number of iterations used with the NCP stopping criteria and the ideal number of iterations is shown for the SIRT-LC algorithm as a function of time step in the simulation.

S8 ℓ_2-Norm Difference Between NCP and Ideal Stopping Criteria

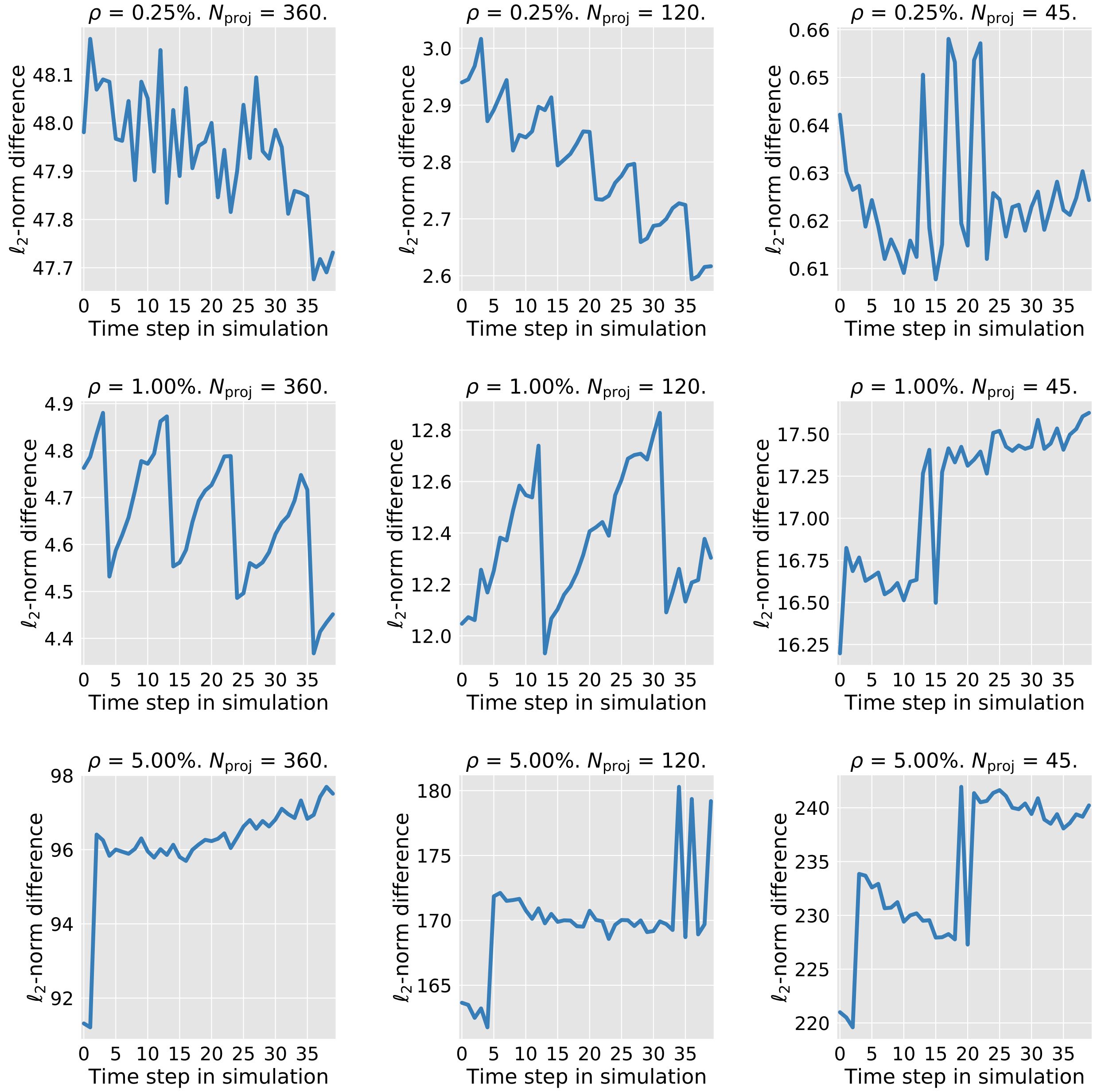

Figure S16: The difference between the ℓ_2-norm of residual when using the NCP stopping criteria and the ideal ℓ_2-norm is shown for the SIRT algorithm as a function of time step in the simulation.

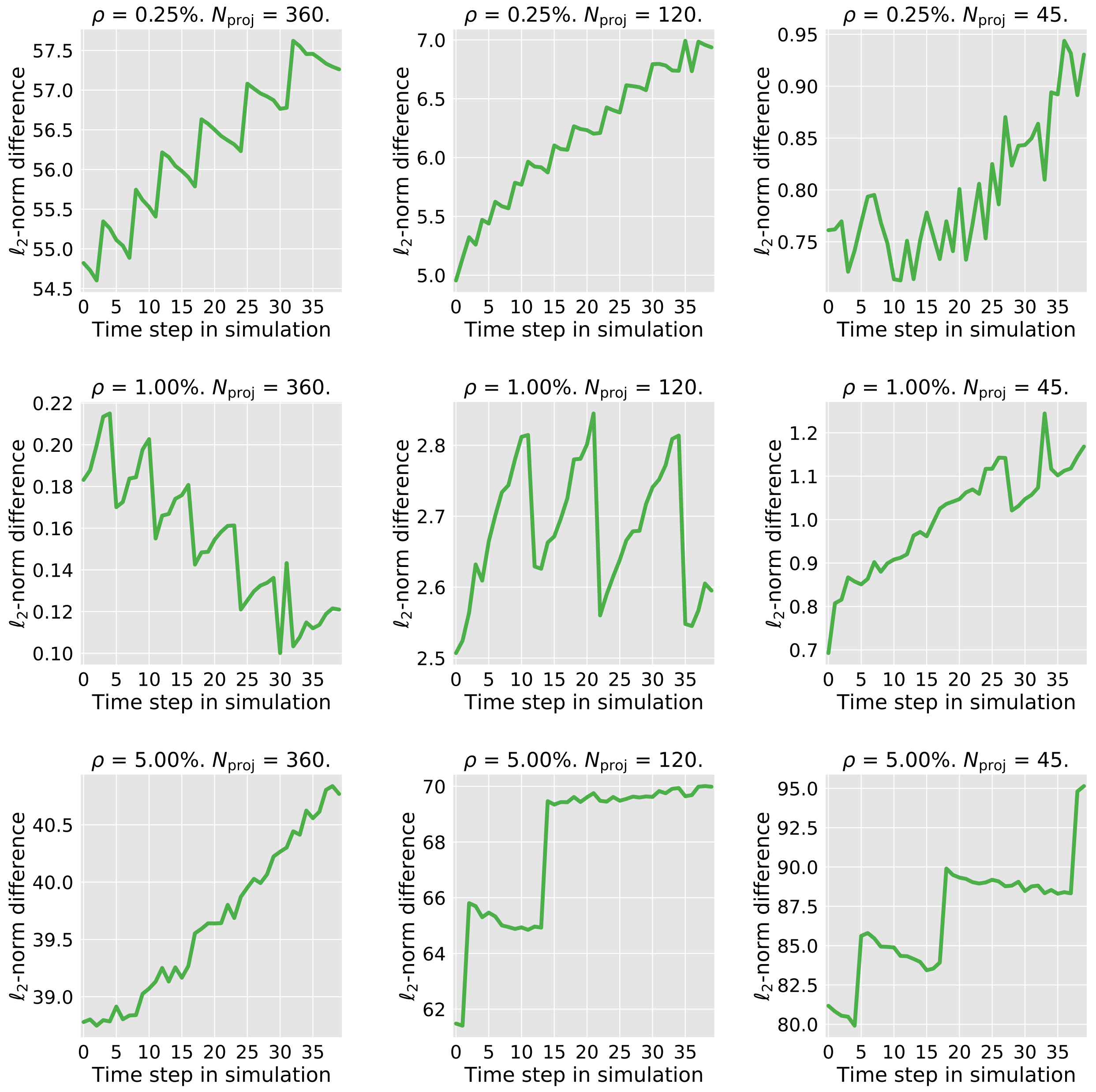

Figure S17: The difference between the ℓ_2-norm of residual when using the NCP stopping criteria and the ideal ℓ_2-norm is shown for the SIRT-BC algorithm as a function of time step in the simulation.

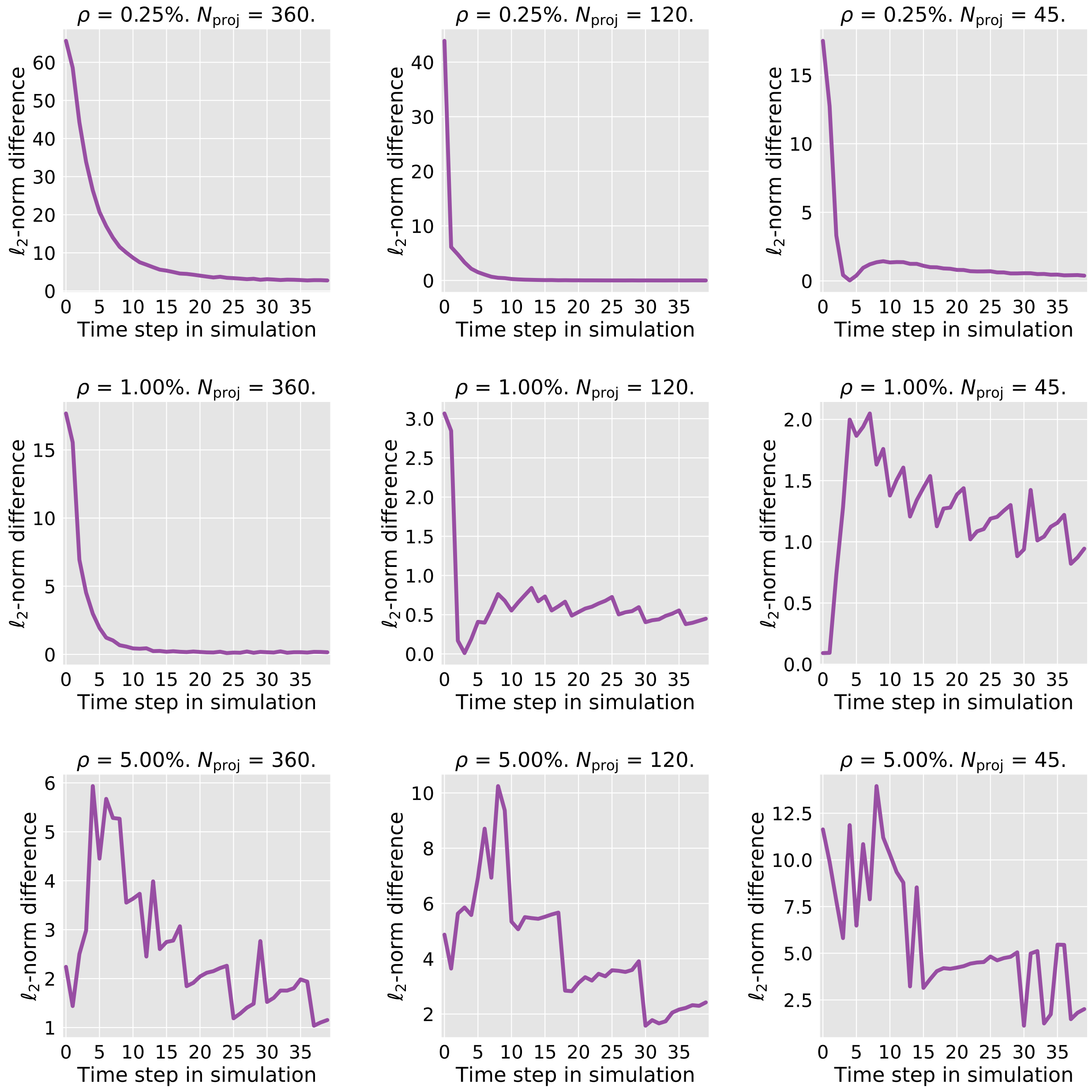

Figure S18: The difference between the ℓ_2-norm of residual when using the NCP stopping criteria and the ideal ℓ_2-norm is shown for the SIRT-IC algorithm as a function of time step in the simulation.

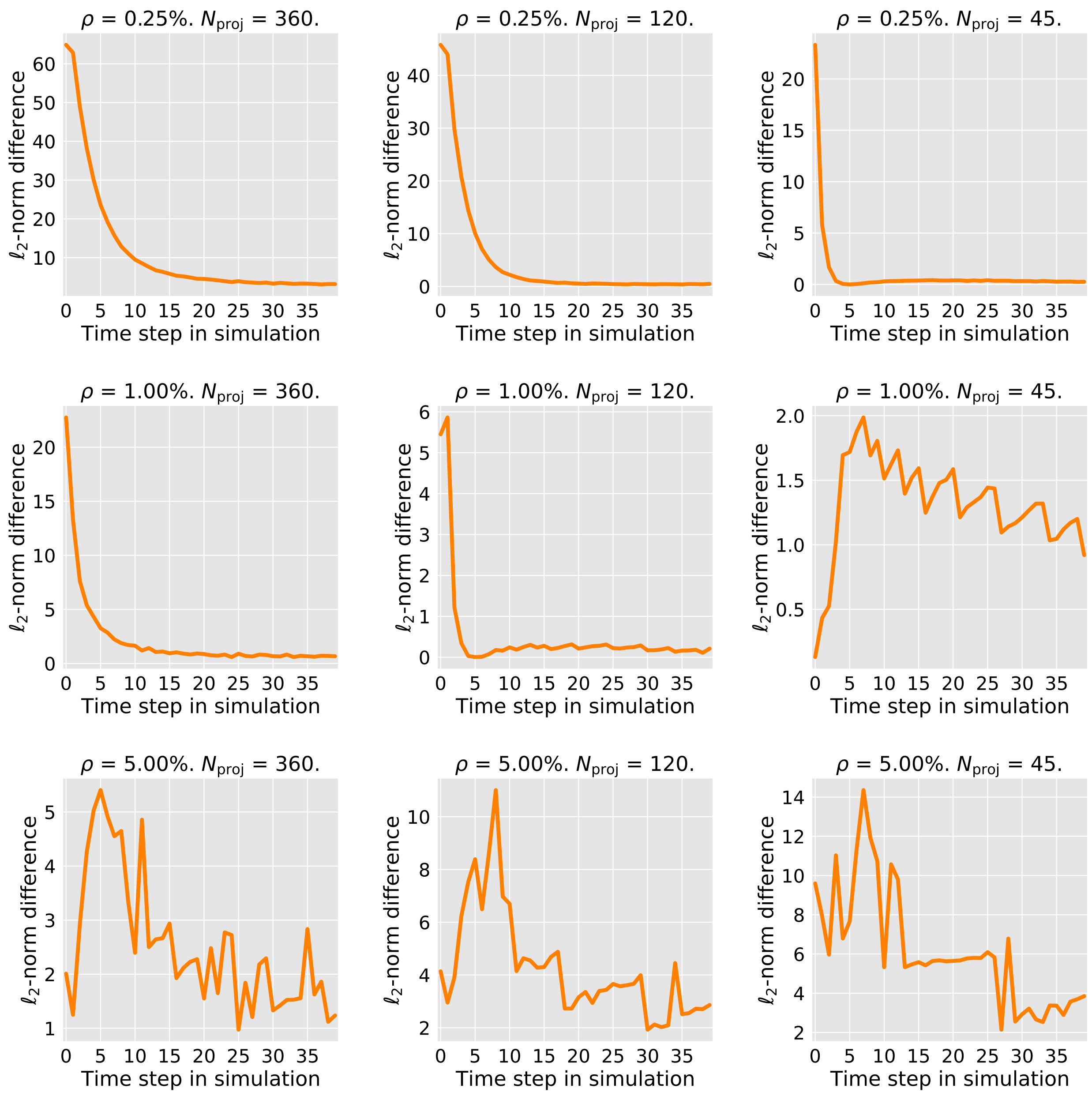

Figure S19: The difference between the ℓ_2-norm of residual when using the NCP stopping criteria and the ideal ℓ_2-norm is shown for the SIRT-LC algorithm as a function of time step in the simulation.

S9 Examples of Residuals of the Reconstructions

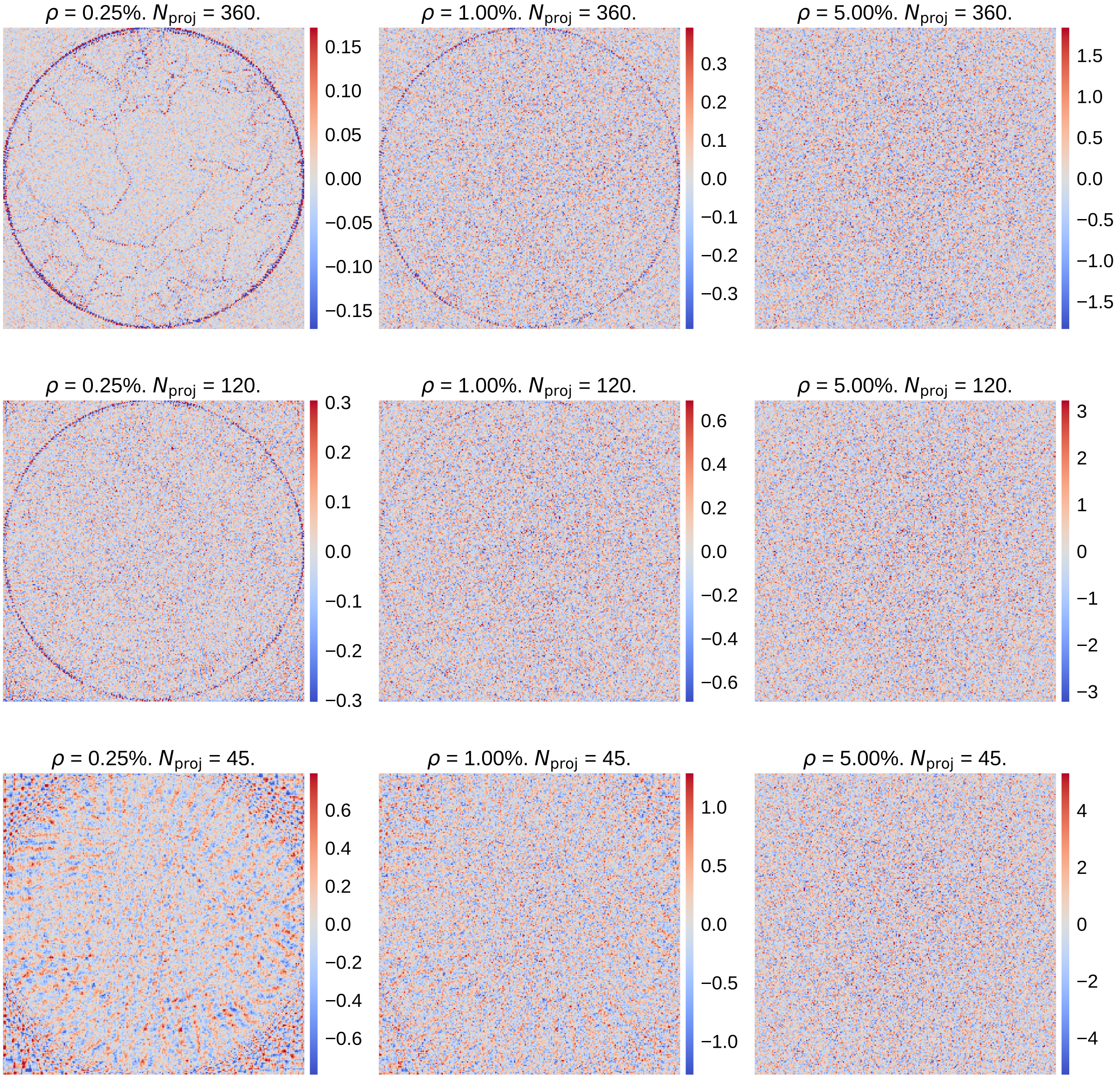

Figure S20: Examples of the residual between the reconstruction and ground truth for all data sets with the FBP algorithm.

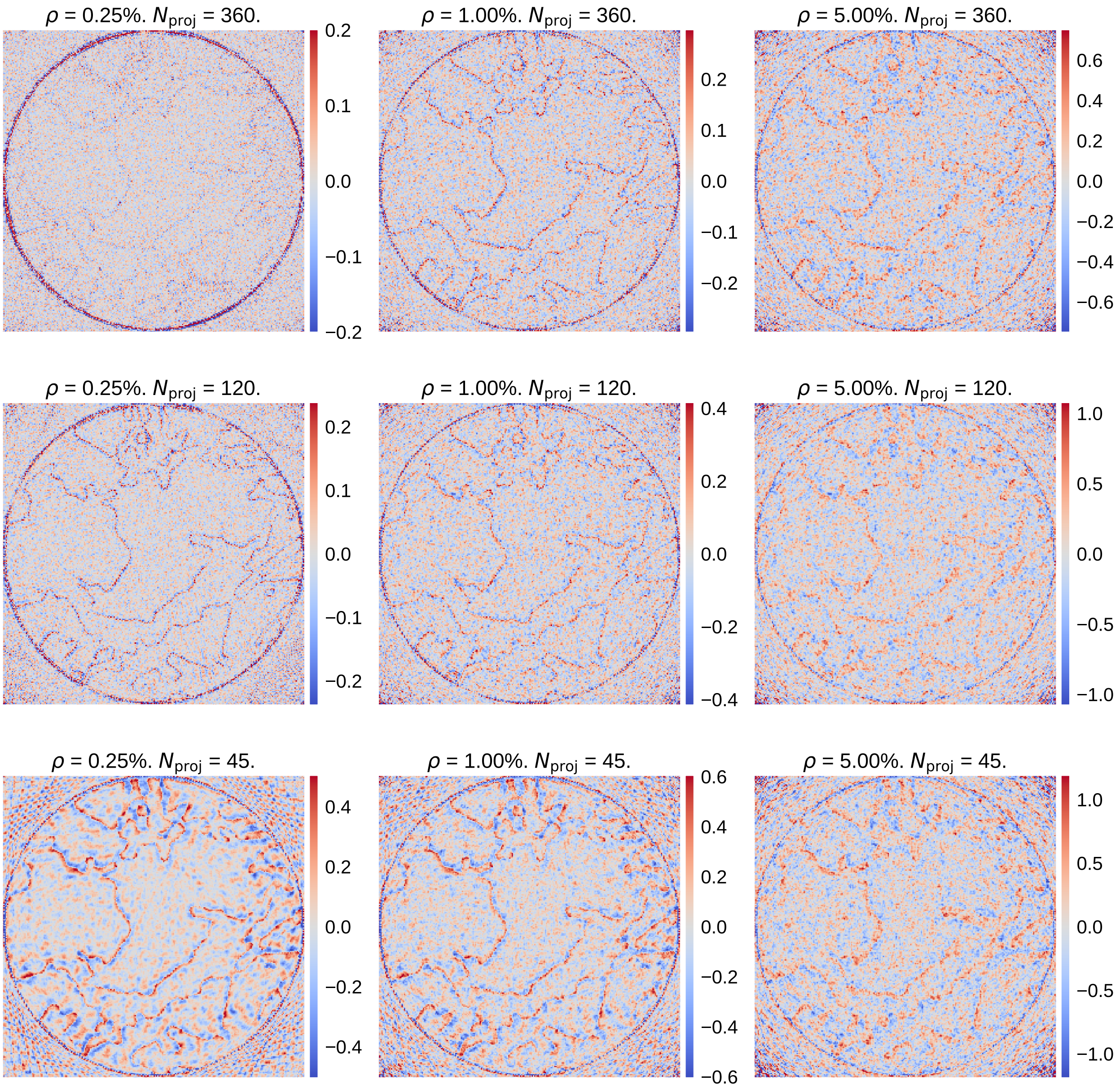

Figure S21: Examples of the residual between the reconstruction and ground truth for all data sets with the SIRT algorithm.

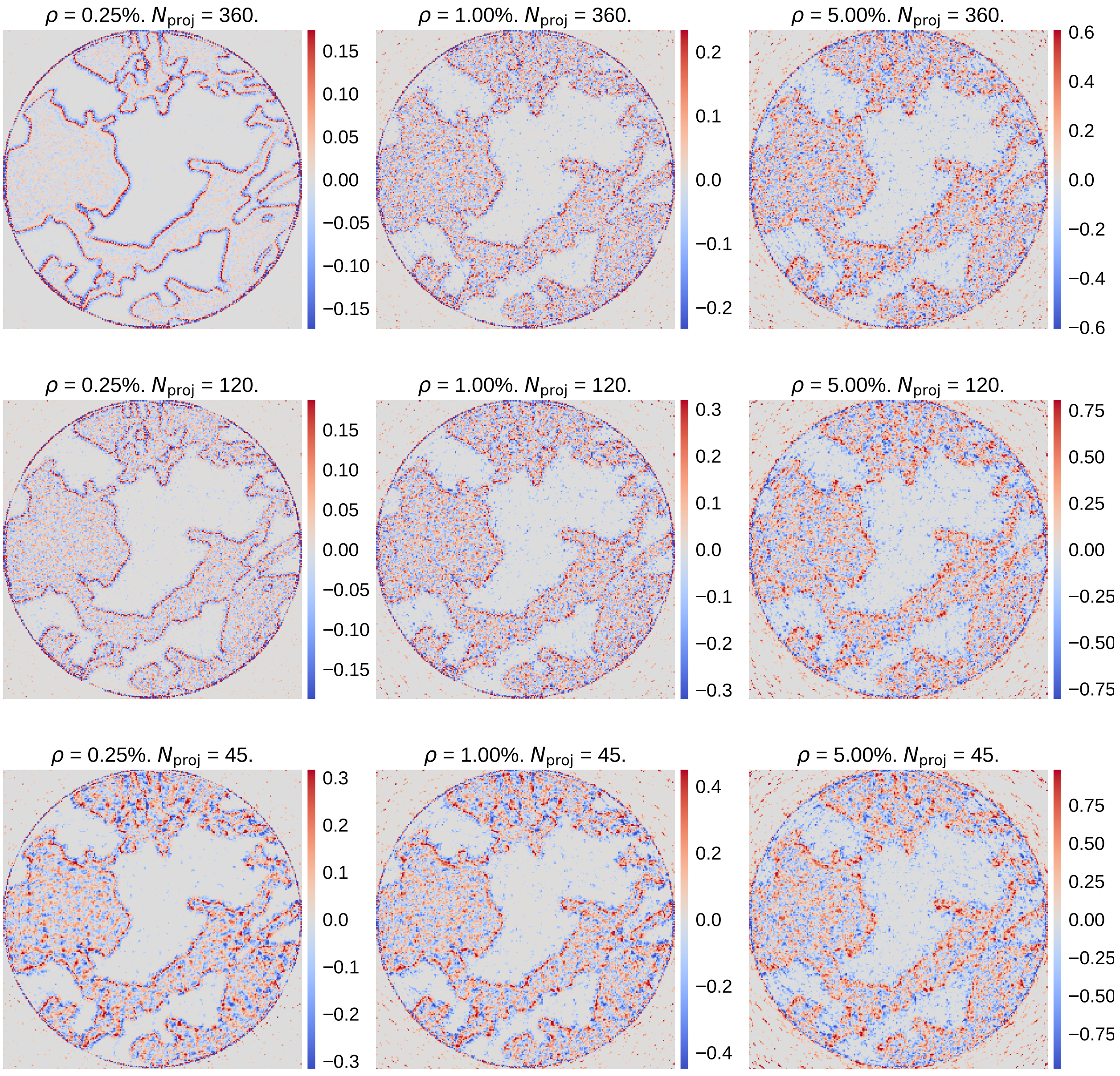

Figure S22: Examples of the residual between the reconstruction and ground truth for all data sets with the SIRT-BC algorithm.

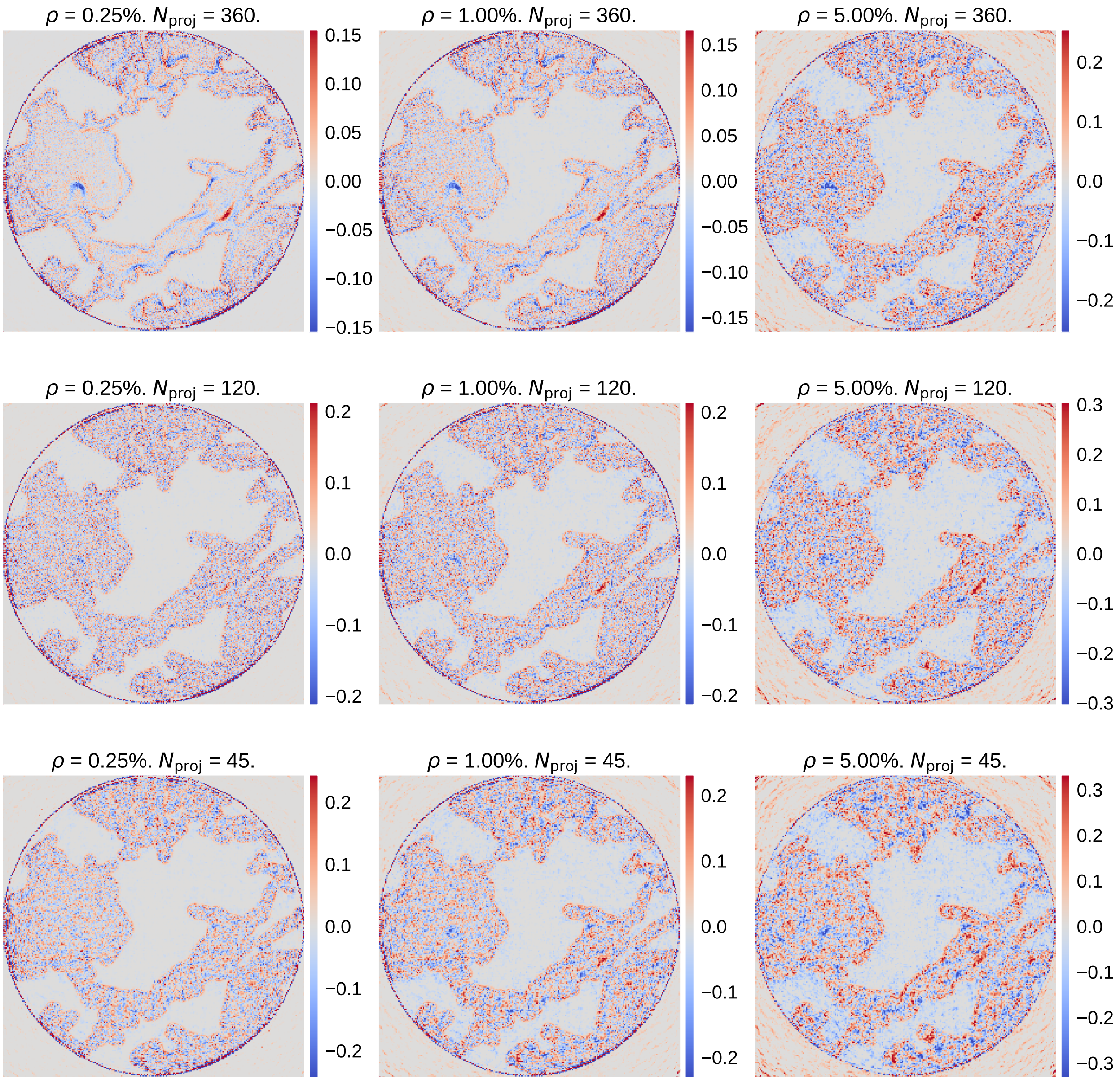

Figure S23: Examples of the residual between the reconstruction and ground truth for all data sets with the SIRT-IC algorithm.

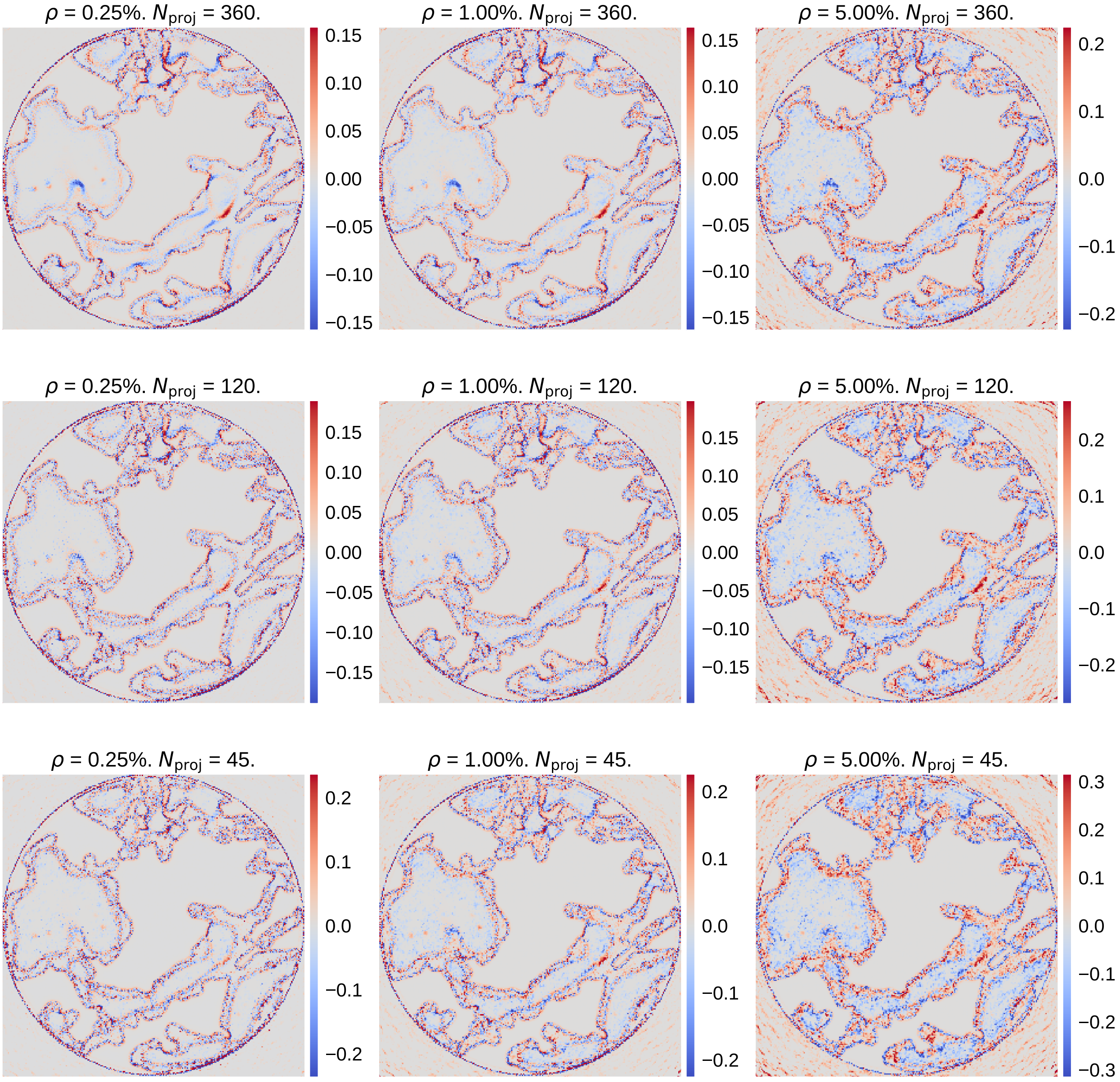

Figure S24: Examples of the residual between the reconstruction and ground truth for all data sets with the SIRT-LC algorithm.

S10 Overall Performance of all Algorithms on all Data Sets

Data	Norm	FBP	SIRT	SIRT-BC	SIRT-IC	SIRT-LC
$N_{\text{proj}} = 360$ $\rho = 0.25\%$	ℓ_1 $(\cdot 10^7)$	4.13	4.76	2.78	2.74	**2.31**
	ℓ_2 $(\cdot 10^3)$	1.51	1.70	1.54	**1.34**	1.43
$N_{\text{proj}} = 360$ $\rho = 1.00\%$	ℓ_1 $(\cdot 10^7)$	12.85	8.15	5.52	3.30	**2.55**
	ℓ_2 $(\cdot 10^3)$	4.52	2.94	2.52	**1.52**	**1.52**
$N_{\text{proj}} = 360$ $\rho = 5.00\%$	ℓ_1 $(\cdot 10^7)$	62.62	21.25	15.56	6.31	**4.31**
	ℓ_2 $(\cdot 10^3)$	22.02	7.52	6.87	2.76	**2.36**
$N_{\text{proj}} = 120$ $\rho = 0.25\%$	ℓ_1 $(\cdot 10^7)$	8.78	5.78	3.78	4.24	**2.95**
	ℓ_2 $(\cdot 10^3)$	3.10	2.15	1.81	1.98	**1.75**
$N_{\text{proj}} = 120$ $\rho = 1.00\%$	ℓ_1 $(\cdot 10^7)$	22.88	11.11	7.82	4.72	**3.32**
	ℓ_2 $(\cdot 10^3)$	8.05	3.98	3.53	2.11	**1.90**
$N_{\text{proj}} = 120$ $\rho = 5.00\%$	ℓ_1 $(\cdot 10^7)$	109.23	27.45	20.89	7.83	**5.33**
	ℓ_2 $(\cdot 10^3)$	38.40	9.69	9.08	3.38	**2.89**
$N_{\text{proj}} = 45$ $\rho = 0.25\%$	ℓ_1 $(\cdot 10^7)$	21.47	12.46	7.19	4.75	**3.73**
	ℓ_2 $(\cdot 10^3)$	7.56	4.62	3.35	**2.20**	**2.20**
$N_{\text{proj}} = 45$ $\rho = 1.00\%$	ℓ_1 $(\cdot 10^7)$	40.74	15.66	10.99	5.26	**3.98**
	ℓ_2 $(\cdot 10^3)$	14.32	5.68	4.98	2.31	**2.24**
$N_{\text{proj}} = 45$ $\rho = 5.00\%$	ℓ_1 $(\cdot 10^7)$	179.59	32.49	25.77	8.68	**6.25**
	ℓ_2 $(\cdot 10^3)$	63.21	11.49	11.06	3.69	**3.34**

Table S1: Table of the ℓ_1- and ℓ_2-norms for all data sets. Bold numbers indicate the best performing algorithm.

S11 Parameters Used for Multiphase Flow Analysis

	Calcite	Water	Oil
Phase value	0.0	0.5	-0.5
Density [$\mathrm{g\,cm^{-3}}$]	-	1.02	0.76
Relaxation parameter	-	0.67	0.80
	Other Parameters		
Contact angle	90°		
Surface tension	$0.0083\ \mathrm{N\,m^{-1}}$		
Interface mobility	0.01		
Interface width	3 [Voxels]		
Collision mode	TRT; Magic number = 1/4		

Table S2: List of parameters used for the multiphase flow simulations.
